# Neonatal immune challenge poses a sex-specific risk for epigenetic microglial reprogramming and behavioral impairment

**DOI:** 10.1038/s41467-023-38373-0

**Published:** 2023-05-11

**Authors:** Marius Schwabenland, Omar Mossad, Annika Sievert, Adam G. Peres, Elena Ringel, Sebastian Baasch, Julia Kolter, Giulia Cascone, Nikolaos Dokalis, Andreas Vlachos, Zsolt Ruzsics, Philipp Henneke, Marco Prinz, Thomas Blank

**Affiliations:** 1 Institute of Neuropathology, Faculty of Medicine, University of Freiburg, Freiburg, Germany; 2 Faculty of Biology, University of Freiburg, Freiburg, Germany; 3 Institute for Immunodeficiency, Center for Chronic Immunodeficiency (CCI), Medical Center, University of Freiburg, Faculty of Medicine, University of Freiburg, Freiburg, Germany; 4 Department of Neuroanatomy, Institute of Anatomy and Cell Biology, Faculty of Medicine, University of Freiburg, Freiburg, Germany; 5 Center for Basics in Neuromodulation (NeuroModulBasics), Faculty of Medicine, University of Freiburg, Freiburg, Germany; 6 Institute for Virology, Faculty of Medicine, Medical Center, University of Freiburg, Freiburg, Germany; 7 Center for Pediatrics and Adolescent Medicine, Medical Center, University of Freiburg, Faculty of Medicine, University of Freiburg, Freiburg, Germany; 8 CIBSS Centre for Integrative Biological Signalling Studies, University of Freiburg, Freiburg, Germany; 9 Signalling Research Centres BIOSS and CIBSS-Centre for Integrative Biological Signalling Studies, University of Freiburg, Freiburg, Germany

**Keywords:** Development of the nervous system, Neuroimmunology

## Abstract

While the precise processes underlying a sex bias in the development of central nervous system (CNS) disorders are unknown, there is growing evidence that an early life immune activation can contribute to the disease pathogenesis. When we mimicked an early systemic viral infection or applied murine cytomegalovirus (MCMV) systemically in neonatal female and male mice, only male adolescent mice presented behavioral deficits, including reduced social behavior and cognition. This was paralleled by an increased amount of infiltrating T cells in the brain parenchyma, enhanced interferon-γ (IFNγ) signaling, and epigenetic reprogramming of microglial cells. These microglial cells showed increased phagocytic activity, which resulted in abnormal loss of excitatory synapses within the hippocampal brain region. None of these alterations were seen in female adolescent mice. Our findings underscore the early postnatal period’s susceptibility to cause sex-dependent long-term CNS deficiencies following infections.

## Abstract (web summary)

Early life inflammation has been linked to neurodevelopmental outcomes. Here the authors show that in mice, neonatal immune challenge results in CD3 + T cell infiltration to brain parenchyma, and microglial activation, to a greater extent in males than females.

## Introduction

Perinatal brain damage is one of the primary causes of lifelong disabilities such as cerebral palsy, seizure disorders, sensory impairment, and cognitive limitations^1^. Emerging data clearly suggests that infection and inflammation are key contributors to the pathogenesis of perinatal brain injury and consequent long-term impairment in brain function^2^. Indeed, a number of developmental disorders, including schizophrenia, autism spectrum disorder (ASD), depressive symptoms and psychotic experiences, have been linked to early life immune activation and subsequent dysregulation of immune function^3–5^. When an inflammatory milieu develops in the central nervous system (CNS), microglia are among the first responders and rapidly obtain an upregulated or activated phenotype^6^. The immune functions of microglia are regulated by cytokines, including interferon-γ (IFNγ), which is a major mediator of microglia activation. As a result, microglia undergo changes in morphology, surface antigen expression and produce numerous pro- and anti-inflammatory cytokines^7,8^. Because microglia shape the neuronal network formation within the neonatal CNS, small changes in microglia activity during this early time period may impair the normal course of brain development. Disturbed microglia function can affect synaptic maintenance^9^ or phagocytosis of living cells, dying or dead cells and axons^10^. Microglial dysfunction might also have an adverse effect on myelination/oligodendrogenesis, neurogenesis and axon fasciculation^11–13^. According to epidemiological statistics, the prevalence of developmental abnormalities, such as ASD, Attention Deficit Hyperactivity Disorder (ADHD) and general learning disabilities is twice as high in boys as it is in girls^14^. As a result, while investigating the genesis of neurodevelopmental diseases connected to early immunological activation, sex has emerged as an important aspect to examine. Despite the significant experimental evidence of the long-term and dramatic influence of early life immunological stressors on neurodevelopmental disorders, we still do not know how these imprinting processes are activated and how long they persist. Microglia in the hippocampus of rats, for example, exhibit a significant rise in *Cd11b* mRNA following a peripheral infection at postnatal day 4, and this increase persists throughout adulthood^15^.

Herein, we report that a neonatal immune challange triggers the entry of CD3^+^ T cells into the brain parenchyma to a larger extent in males than in females. The higher number of invading CD3^+^ T cells in males was associated with significantly elevated IFNγ brain levels. IFNγ elicited sustained epigenetic changes in male microglia, which showed augmented pruning of excitatory synapses that worsened hippocampal brain function and behavior during adolescence. In contrast, microglia were only transiently activated after early immune stimulation in female mice. Since human neurodevelopmental disorders often show a sex bias^16^, the observed sex differences in epigenetic microglia programming following early immune activation may be of central importance.

## Results

### Neonatal immune activation drives chronic behavioral deficits and microglial activation in males

**Fig. 1:**
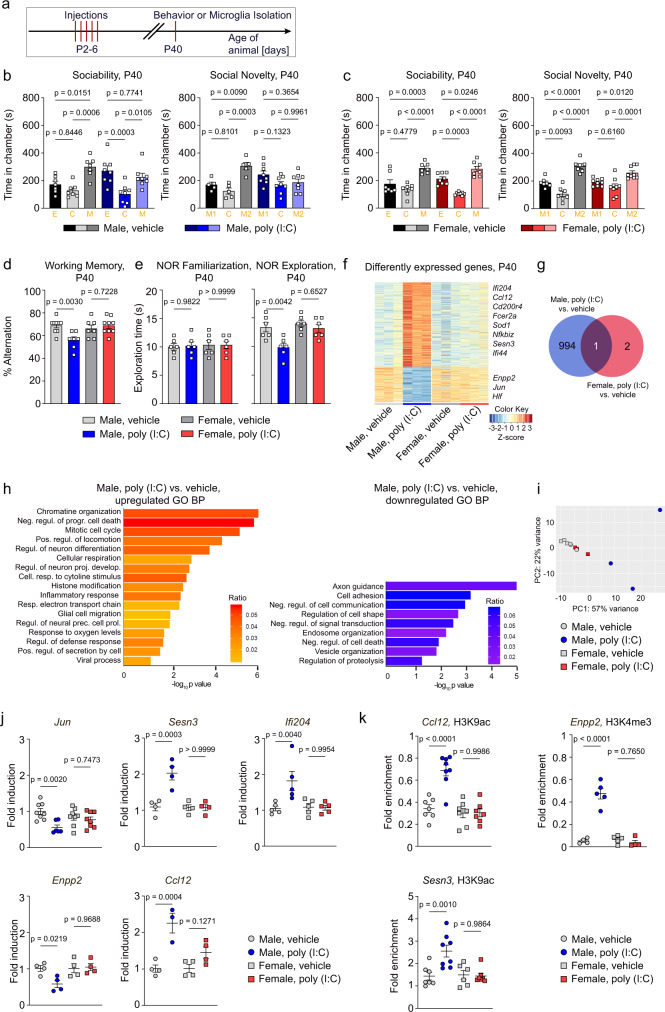
Poly (I:C)-induced behavioral deficits are accompanied by transcriptional and epigenetic changes in microglia of male mice. **a** Scheme of the experimental timeline. **b** Left panel: sociability of male mice was measured at P40 by performing the three-chamber test: time spent by vehicle- and poly (I:C)-injected male mice in one of the three compartments of the test apparatus: E (empty), C (center), M (mouse). Preference for interacting with the mouse (M) over the empty compartment (E) as seen in the controls could not be observed in poly (I:C)-injected male mice. Data are presented as mean ± SEM. Each color-coded symbol represents data of an individual mouse (*n* = 7 for Male, vehicle; *n* = 8 for Male, poly (I:C)). Significant differences were determined by one-way ANOVA followed by Sidak multiple comparison test. *P* values are provided in the figure. Right panel: social novelty was measured by conducting the three-chamber test with a familiar mouse (M1), an empty compartment (E) and a stranger mouse (M2). Preference for the stranger mouse (M2) over the familiar mouse (M1) as seen in control mice could not be observed in male poly (I:C)-injected mice. Each color-coded symbol represents data of an individual mouse (*n* = 5 for Male, vehicle; *n* = 8 for Male, poly (I:C)). Significant differences were determined by one-way ANOVA followed by Sidak multiple comparison test. *P* values are provided in the figure. **c** Left panel: sociability of female mice was measured at P40 by conducting the three-chamber test: time spent by female vehicle-injected and poly (I:C)-injected mice in the three compartments of the test apparatus: E (empty), C (center), M (mouse). Preference for the mouse (M) over the empty compartment (E) was observed in both, vehicle- and poly (I:C)- injected female mice. Data are presented as mean ± SEM. Each color-coded symbol represents data of an individual mouse (*n* = 7 for Female, vehicle; *n* = 8 for Female, poly (I:C)). Significant differences were determined by one-way ANOVA followed by Sidak multiple comparison test. *P* values are provided in the figure. Right panel: social novelty was measured by conducting the three-chamber test with a familiar mouse (M1), an empty compartment (E) and a stranger mouse (M2). Preference for the stranger mouse (M2) over the familiar mouse (M1) was observed in both vehicle- and poly (I:C)-injected female mice. Data are presented as mean ± SEM. Each color-coded symbol represents data of an individual mouse (*n* = 7 for Female, vehicle; *n* = 9 for Female, poly (I:C)). Significant differences were determined by one-way ANOVA followed by Sidak multiple comparison test. P values are provided in the figure. **d** Percentage alteration in a T-maze test showing spatial working memory defects in poly (I:C)-injected mice compared to vehicle-injected mice. Data are presented as mean ± SEM. Each color-coded symbol represents data of an individual mouse (*n* = 9 for Male, vehicle; *n* = 7 for Male, poly (I:C); *n* = 7 for Female, vehicle; *n* = 9 for Female, poly (I:C)). Significant differences were determined by one-way ANOVA followed by Sidak multiple comparison test. *P* values are provided in the figure. **e** Left panel: exploration time in the novel object recognition test during familiarization. Right panel: exploration time of a novel object in the novel object recognition test. Data are presented as mean ± SEM. Each color-coded symbol represents data of an individual mouse (*n* = 6 per group). Significant differences were determined by one-way ANOVA followed by Sidak multiple comparison test. *P* values are provided in the figure. **f** Heat map of differentially expressed microglial genes at P40 from male and female control and poly (I:C)-treated mice, determined by RNA-Seq analysis of isolated microglia. Genes sorted by log_2_ fold change in descending order. Signals are scaled to Z-scores of the rows. The colored bar below the heat map (horizontal dimension) indicates the grouping variable. **g** The Venn diagram illustrates the overlap between differentially expressed microglial genes from poly (I:C)-treated male and female mice. **h** Results of gene ontology (GO) biological process (BP) enrichment analysis associated with downregulated genes (blue color coding) and upregulated genes (orange color coding). **i** Principal component analysis (PCA) of the RNA-Seq data depicted as heat map under (F) for differentially expressed microglial genes at P40. Data obtained from vehicle-treated male (filled circles, gray) and female mice (filled squares, gray) as well as from poly (I:C)-treated male (filled circles, blue) and female mice (filled squares, red) are shown. **j** Validation of RNA-Seq data by quantitative RT-qPCR analysis of indicated genes in isolated microglia from male and female control and poly (I:C)-treated mice. Each color-coded symbol represents data of an individual mouse (*Jun*: *n* = 8 for Male, vehicle; *n* = 6 for Male, poly (I:C); *n* = 8 for Female, vehicle; *n* = 8 for Female, poly (I:C). *Sesn3*: *n* = 4 per group. *Ifi204*: *n* = 5 per group. Enpp2: n = 4 per group. *Ccl12*: *n* = 4 for Male, vehicle; *n* = 3 for Male, poly (I:C); *n* = 4 for Female, vehicle; *n* = 4 for Female, poly (I:C)). Significant differences were determined by one-way ANOVA followed by Sidak multiple comparison test. *P* values are provided in the figure. **k** ChIP-qPCR analysis at P40 for H3K4me3 or H3K9ac occupancy on *CCl12, Enpp2* and *Sesn3* promoters in microglia isolated from poly (I:C)- or vehicle-injected male and female mice. Data are expressed as percent of input and normalized to *Gapdh*. Single dots represent the data from one single mouse (*Ccl12*: *n* = 7 for Male, vehicle; *n* = 8 for Male, poly (I:C); *n* = 7 for Female, vehicle; *n* = 7 for Female, poly(I:C). *Enpp2*: *n* = 4 for Male, vehicle; *n* = 5 for Male, poly (I:C); *n* = 5 for Female, vehicle; *n* = 4 for Female, poly(I:C). *Sesn3*: *n* = 7 for Male, vehicle; *n* = 8 for Male, poly (I:C); *n* = 6 for Female, vehicle; *n* = 7 for Female, poly(I:C).). Significant differences were determined by one-way ANOVA followed by Sidak multiple comparison test. *P* values are provided in the figure. Source data are provided as a Source data file.

We first compared the consequences of neonatal immune activation in male and female mice by injecting a low dose of polyriboinosinic:polyribocytidilic acid (poly(I:C)) daily from postnatal days 2 to 6 (P2-6) intraperitoneally (i.p.) (Fig. 1a). Poly (I:C) is a synthetic analog of double-stranded RNA which induces a cytokine-associated, viral-like acute-phase response^17^. In humans, immune stimulation, especially in early development, has been associated with reduced social behavior^18^, impaired working memory profiles^19^ and diminished recognition memory^20^. Our goal was now to reproduce the behavioral changes shown in humans in our mouse model and evaluated potential behavioral abnormalities at peripubertal age (P40). The reason for choosing this age of the animals was because the onset of many behavioral disorders occurs during this developmental stage^21^. When comparing mice of both sexes, only poly (I:C)-treated male mice showed impaired social behavior as displayed in the three-chamber test (Fig. 1b, c), defective spatial working memory (T-maze test, Fig. 1d) and diminished recognition memory (novel-object recognition test (NOR), Fig. 1e) when compared to their vehicle-treated male controls. Mice of both sexes and both treatment groups showed similar behavior during familiarization with the NOR (Fig. 1e). We further investigated the effects of poly (I:C)-injected pups on the overall development at day P7, P15 and P40. First, we observed a reduction in body weight (Fig. S1a) and in brain weight (Fig. S1b) in pups of both sexes subsequent to the poly (I:C) treatment when compared to the control groups. While the body weight remained lower in the poly (I:C)-treated group for both sexes at P40, the brain weight was significantly lower only in males relative to male controls. In female mice, the brain weight of control and poly (I:C)-treated mice was identical at P40 (Fig. S1b).

The observed behavioral changes might be due to the fact that neonatal immune activation alters immunocompetent cells in the CNS, which are subsequently proficient to modulate brain function. We first used molecular techniques to study changes in microglia, a population of cells which share both of these features^7,22^. In order to analyze the expression pattern of genes affected by early immune stimulation, we performed RNA-sequencing analysis (RNA-Seq) on microglial cells isolated from male and female mice at P40 (Fig. 1f). The isolated cell populations were of high purity, based on the expression analysis revealing almost exclusively microglial genes with only minor contribution of genes expressed by endothelial, neuronal, astrocytic, oligodendrocytic and oligodendrocytic precursor cells (Fig. S1c, Supplementary Dataset 1). While in poly (I:C)-treated males 995 genes were differentially regulated, there were only 3 genes differentially regulated in female mice in comparison to vehicle-treated controls (Fig. 1g, Supplementary Dataset 2). One of the differentially regulated genes was overlapping. Functional annotation of the differentially regulated genes revealed that the clusters of up-regulated genes in poly (I:C)-treated male mice included genes related to chromatin organization, histone modification, inflammation, defense and viral processes. On the other hand, the biological processes associated with down-regulated genes were related to cell adhesion, endosome organization, cell death and proteolysis (Fig. 1h). The respective PCA diagram showed that male and female controls as well as female poly (I:C)-treated samples clustered together while male poly (I:C)-treated samples clustered separately (Fig. 1i).

We confirmed the differential gene expression in immune-challenged male mice using RT-qPCR against some of the top differentially expressed genes (Fig. 1j). The expression of *Jun proto-oncogene* (*Jun*) and of *ectonucleotide pyrophosphatase/phosphodiesterase 2* (*Enpp2*) was downregulated in the poly (I:C) group, which should result in anti-inflammatory actions in microglia^23,24^. Enhanced expression was detectable for *C-C motif chemokine 12* (*Ccl12*), *interferon-inducible gene 204* (*Ifi204*) and *sestrin 3* (*Sesn3*), which are all related to microglia activation. Differential gene expression was only present in immune-challenged male mice while the gene expression in female mice remained unchanged.

We hypothesized that these longerlasting transcriptomic changes in microglia are the result of epigenetic processes. To test this hypothesis, we analyzed H3K9 acetylation (ac) and H3K4 trimethylation (me3) histone modifications by chromatin immunoprecipitation quantitative PCR (ChIP-qPCR) of in microglia isolated from P40 mice that were neonatally challenged with poly (I:C), or their corresponding littermate controls (Fig. 1k). Here, we focused on *Ccl12*, *Sesn3,* and *Enpp2*, which had all shown a differential gene expression in response to poly (I:C). We followed up on those three genes because upregulation of *Ccl12* is indicative of an inflammatory gene expression profile^25^. *Sesn3* knockdown in microglia cells was shown to decrease the expression of proinflammatory mediators, indicating the important role of *Sens3* in orchestrating proinflammatory responses^26^. Upregulation of Autotaxin, the gene product of *Enpp2*, inhibits microglia activation and prevents cytokine production^27^. *Ccl12* and *Sesn3* showed increased H3K9ac binding levels while *Enpp2* displayed higher H3K4me3 binding levels. These alterations were seen in microglia from immune-stimulated male mice when compared to microglia from vehicle-treated mice and were mirrored by enhanced gene expression of *Ccl12* and *Sesn3* and downregulation of *Enpp2* (Fig. 1j). Changes in histone modifications after poly (I:C) injection were missing in microglia isolated from female mice (Fig. 1k), which corresponded to steady gene expression levels between vehicle- and poly (I:C)-treated groups (Fig. 1j). Collectively, these data indicate that early immune stimulation, particularly in male mice, changes the acetylation and methylation marks of defined gene promoter regions in microglia.

### Only microglia of male immune-challenged mice modify newly generated neurons in the dentate gyrus

**Fig. 2:**
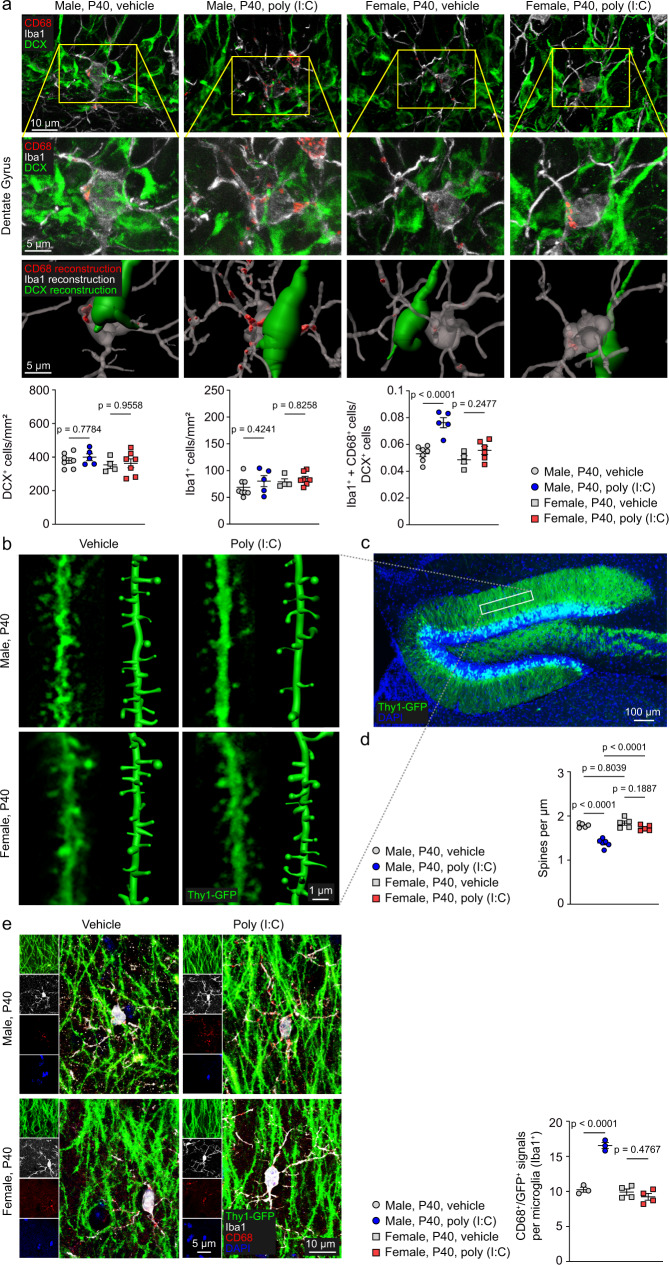
Activated microglia of male poly (I:C)-treated mice display sustained disturbance of the neuronal connectivity within the dentate gyrus. **a** Upper lane: immunohistochemical detection of CD68 (red), Iba1 (white), DCX (green) and DAPI (blue) at postnatal day 40 in the dentate gyrus of male and female poly (I:C)- and vehicle-treated mice. Scale bar = 10 µm. Middle lane: higher magnification of the images shown in the upper lane. Scale bar = 5 µm. Lower lane: representative 3D reconstruction based on the immunofluorescent images shown in the middle lane. DCX is reconstructed in green, CD68 in red, Iba1 in transparent white. The Iba1 reconstruction is shown in a translucent mode, allowing the reconstructed CD68 signal to shine through with different intensities depending on the intracellular localization. Scale bar = 5 µm. Graphs below the images depict the quantification of DCX^+^ (*n* = 7 for Male, vehicle; *n* = 5 for Male, poly (I:C); *n* = 4 for Female, vehicle; *n* = 7 for Female, poly (I:C)), Iba1^+^ (*n* = 8 for Male, vehicle; *n* = 5 for Male, poly (I:C); *n* = 4 for Female, vehicle; *n* = 7 for Female, poly (I:C)) and Iba1^+^+CD68^+^ cells per DCX^+^ cell (*n* = 7 for Male, vehicle; *n* = 5 for Male, poly (I:C); *n* = 4 for Female, vehicle; *n* = 6 for Female, poly (I:C)) in the dentate gyrus. Each symbol represents one mouse. Data are presented as mean ± SEM. Significant differences were determined by one-way ANOVA followed by Sidak multiple comparison test. *P* values are provided in the figure. **b** Fluorescent images of Thy1-GFP^+^ cells in vehicle-treated and poly (I:C)-treated animals of both sexes. Representative 3D reconstructions of dendritic spines are shown next to the original histological images. Scale bar = 1 µm. The area of interest is shown in (**c**) and marked with a white rectangle. Scale bar = 100 µm. Quantification of dendritic spines in the indicated region within the dentate gyrus is shown in (**d**). Each symbol represents one mouse (*n* = 5 for Male, vehicle; *n* = 6 for Male, poly (I:C); *n* = 5 for Female, vehicle; *n* = 5 for Female, poly (I:C)). Data are presented as mean ± SEM. Significant differences were determined by one-way ANOVA followed by Sidak multiple comparison test. *P* values are provided in the figure. **e** Immunofluorescence staining in P40 mice of Iba1 (white), CD68 (red) and DAPI (blue) in vehicle- and poly (I:C)-injected Thy1-GFP (green) male and female animals (left). Scale bars = 10 µm in the merged images and 5 µm in the inserts. Quantification of CD68^+^/ Thy1-GFP^+^ signals per Iba1^+^ microglia is shown (right). Each symbol represents one mouse (*n* = 3 for Male, vehicle; *n* = 3 for Male, poly (I:C); *n* = 4 for Female, vehicle; *n* = 4 for Female, poly (I:C)). Data are presented as mean ± SEM. Significant differences were determined by one-way ANOVA followed by Sidak multiple comparison test. *P* values are provided in the figure. Source data are provided as a Source data file.

Since the behaviors tested in this study are largely dependent on hippocampal function involving the dentate gyrus (DG)^28–30^, we used immunohistochemical techniques to study the activation of microglia in this region of the brain. Quantification of microglia immunoreactive for the calcium-binding protein Iba1 revealed that neonatal poly (I:C) exposure had no effect on total microglia numbers in the DG at the adolescent stage (P40) in males and females (Fig. 2a). In males, the exposure to neonatal immune activation increased the expression of microglial CD68 (Fig. 2a), a lysosomal marker typically expressed by activated microglia in the CNS^31^. The same treatment did not alter CD68 signals in female mice. Three-dimensional reconstruction showed that the CD68^+^ signal was closely localized to immature, doublecortin (DCX)-positive hippocampal neurons^32^ whose number did not change across all groups (Fig. 2a). Moreover, the rate of proliferation and cell death of immature neurons, as indicated by the proliferation marker Ki67 and by positive signals for actived caspase 3, respectively, was similar for all investigated groups at P40 (Fig. S2a, b). Activated microglia have been proposed to reduce the number of dendritic spines by pruning or nibbling^33^. The higher number of activated microglia in the DG of male poly (I:C)-treated mice corresponded with a lower number of spines found on neurons of the DG in these mice at P40 (Fig. 2b–d). During this pruning period at around P40, we used high resolution confocal imaging to assess the interactions between microglia and synaptic inputs in the DG (Fig. 2e). We detected more GFP-labeled dendritic spines within the lysosome (defined by the CD68 signal) of microglia in male mice, which had experienced neonatal immune-stimulation, in comparison to vehicle-treated controls (Fig. 2e). In some cases, the GFP signal in lysosomal structures was already disconnected from any neighboring neuronal processes (Fig. S2c). The occurrence of enhanced pruning present in male mice following immune stimulation was absent in female mice.

### Distinctive IFNγ level in CNS and periphery of neonatal male and female mice after poly (I:C) exposure

**Fig. 3:**
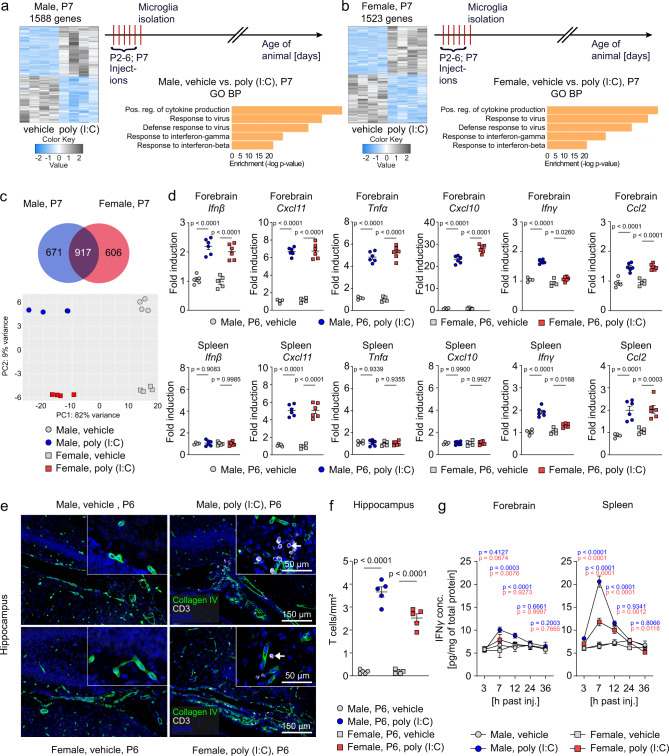
Male mice show a more pronounced acute response to early immune stimuli than female mice. **a**, **b** Upper right panel: schematic diagram of the experimental timeline. Left panel: Heat map of mRNA expression values determined by RNA-Seq in microglia isolated at P7 from forebrain of male (A) and female (B) vehicle- and poly (I:C)-treated mice (*n* = 4,4 males; *n* = 4,4 females). Heatmap displays z-transformed expression values. The most significant terms acquired from gene ontology (GO) enrichment analysis: Biological Procesess (BP) are shown in the lower right panel. **c** Upper panel: The Venn diagram illustrates the overlap between differentially expressed microglial genes from poly (I:C)-treated male and female P7 mice. Lower panel: principal component analysis (PCA) of RNA-Seq data shown in Fig. 3a, b. Data obtained from vehicle-treated male (filled circles, gray) and female mice (filled squares, gray) as well as from poly (I:C)-treated male (filled circles, blue) and female mice (filled squares, red) are shown. **d** Quantitative RT-qPCR of forebrain and spleen lysates obtained 3 h after the last vehicle or poly (I:C) injection at postnatal day 6. Data are expressed as the ratio of gene expression normalized to *Gapdh*. Each color-coded symbol represents data of an individual mouse. Data are presented as mean ± SEM (Forebrain, *Ifnβ*: *n* = 5 for Male, vehicle; *n* = 6 for Male, poly (I:C); *n* = 5 for Female, vehicle; *n* = 6 for Female, poly (I:C). Forebrain, *Cxcl11*: *n* = 4 for Male, vehicle; *n* = 6 for Male, poly (I:C); *n* = 5 for Female, vehicle; *n* = 6 for Female, poly (I:C). Forebrain, *Tnfα*: *n* = 4 for Male, vehicle; *n* = 6 for Male, poly (I:C); *n* = 5 for Female, vehicle; *n* = 6 for Female, poly (I:C). Forebrain, *Cxcl10*: *n* = 4 for Male, vehicle; *n* = 6 for Male, poly (I:C); *n* = 5 for Female, vehicle; *n* = 6 for Female, poly (I:C). Forebrain, *IFNγ*: *n* = 4 for Male, vehicle; *n* = 6 for Male, poly (I:C); *n* = 5 for Female, vehicle; *n* = 6 for Female, poly (I:C). Forebrain, *Ccl2*: *n* = 4 for Male, vehicle; *n* = 6 for Male, poly (I:C); *n* = 5 for Female, vehicle; *n* = 6 for Female, poly (I:C). Spleen, *Ifnβ*: *n* = 4 for Male, vehicle; *n* = 6 for Male, poly (I:C); *n* = 5 for Female, vehicle; *n* = 6 for Female, poly (I:C). Spleen, *Cxcl11*: *n* = 5 for Male, vehicle; *n* = 6 for Male, poly (I:C); *n* = 5 for Female, vehicle; *n* = 6 for Female, poly (I:C). Spleen, *Tnfα*: *n* = 4 for Male, vehicle; *n* = 6 for Male, poly (I:C); *n* = 5 for Female, vehicle; *n* = 6 for Female, poly (I:C). Spleen, *Cxcl10*: *n* = 4 for Male, vehicle; *n* = 6 for Male, poly (I:C); *n* = 5 for Female, vehicle; *n* = 6 for Female, poly (I:C). Spleen, *IFNγ*: *n* = 4 for Male, vehicle; *n* = 6 for Male, poly (I:C); *n* = 5 for Female, vehicle; *n* = 6 for Female, poly (I:C). Spleen, *Ccl2*: *n* = 4 for Male, vehicle; *n* = 6 for Male, poly (I:C); *n* = 5 for Female, vehicle; *n* = 6 for Female, poly (I:C)). Significant differences were determined by one-way ANOVA followed by Sidak multiple comparison test. P values are provided in the figure. **e** Immunohistochemical detection of CD3 (white), collagen IV (green) and DAPI (blue) in the hippocampus of poly (I:C)- or vehicle-injected animals at postnatal day 6. Scale bars = 150 μm, 50 μm (insets). Quantification of parenchymal CD3^+^ cells shown in figure (**e**) is depicted in figure (**f**). Each color-coded symbol represents one mouse (*n* = 5 per group). Data are presented as mean ± SEM. Significant differences were determined by one-way ANOVA followed by Sidak multiple comparison test. *P* values are provided in the figure. **g** IFNγ concentration as measured by enzyme-linked immunosorbent assay (ELISA) in forebrain and spleen lysates at the indicated timepoints after the last injection at postnatal day 6. Each color-coded symbol represents the mean of 5 mice. Data are presented as mean ± SD. For each timepoint, significant differences were determined by one-way ANOVA followed by Sidak multiple comparison test. *P* values for the comparison of male poly (I:C)-treated vs. male vehicle-treated animals are depicted in blue. *P* values for the comparison of female poly (I:C)-injected vs. vehicle-injected animals are shown in red. Source data are provided as a Source data file.

In order to determine the distinctive factors, which are induced during early response in male and female mice, we evaluated the transcriptome of microglia isolated on day P7. We found a similar number of differentially regulated genes and identified the same enriched Gene Ontology (GO) terms for biological processes (BP) in male and female mice (Fig. 3a, b). Out of the approximately 1500 differentially regulated genes, male and female mice shared 917 genes (Fig. 3c, upper image). PCA diagram showed that all samples from male and female controls and poly (I:C)-treated animals clustered separately. PC1 (driven by the poly (I:C) treatment) accounted for 82% and PC2 (driven by the sex of the animals) for 9% of the total variance (Fig. 3c, lower image). Next, we verified typical gene expression changes associated with a poly (I:C) challenge^34,35^ by RT-qPCR using forebrain and splenic tissue on day P6. Expression of genes encoding for *interferon beta* (*IFNβ*) and the proinflammatory cytokines *C-X-C motif chemokine ligand 11* (*Cxcl11*), *tumor necrosis factor alpha* (*Tnfα*), *C-X-C motif chemokine ligand 10* (*Cxcl10*) and *C-C motif chemokine ligand 2* (*Ccl2*) were equally regulated in males and females in response to poly (I:C) (Fig. 3d). The only exception was interferon gamma (*IFNγ*), which was more upregulated in the forebrain and spleen of males than in females following poly (I:C) application (Fig. 3d). Poly (I:C) administration did not cause any downregulation of the *Toll-like receptor-3* (*Tlr3*) mRNA in the spleen (Supplementary Fig. 3a). Because T cells can enter the brain in response to immune stimulations and are further capable of producing IFNγ^36,37^ we quantified parenchymal T cells in the hippocampal brain region of neonatal male and female mice. In order to verify the presence of T cells within the brain parenchyma we performed triple immunostainings which allowed us to identify CD3^+^ T cells with respect to basal laminae proteins (stained with collagen IV) (Fig. 3e). On day P6, the number of CD3^+^ T cells present in the hippocampal parenchyma was higher in brain tissue from poly (I:C)-injected male mice than in brain tissue from poly(I:C)-injected female mice (Fig. 3e, f). An enzyme-linked immunosorbent assay (ELISA) from forebrain and splenic tissue obtained between 3 and 36 h after the last poly (I:C) injection demonstrated significantly higher levels of IFNγ in males than in females with a peak at 7 h after the last poly (I:C) injection (Fig. 3g). In acutely isolated neonatal microglia from male and female mice we found identical levels of phosphorylated STAT1 (pSTAT1) upon IFNγ stimulation (Fig. S3b, c). From this data, we can exclude the possibility that the sensitivity of male and female microglia to IFNγ is different.

### Behavioral deficits and microglial activation in males after neonatal immune activation are T cell-dependent

**Fig. 4:**
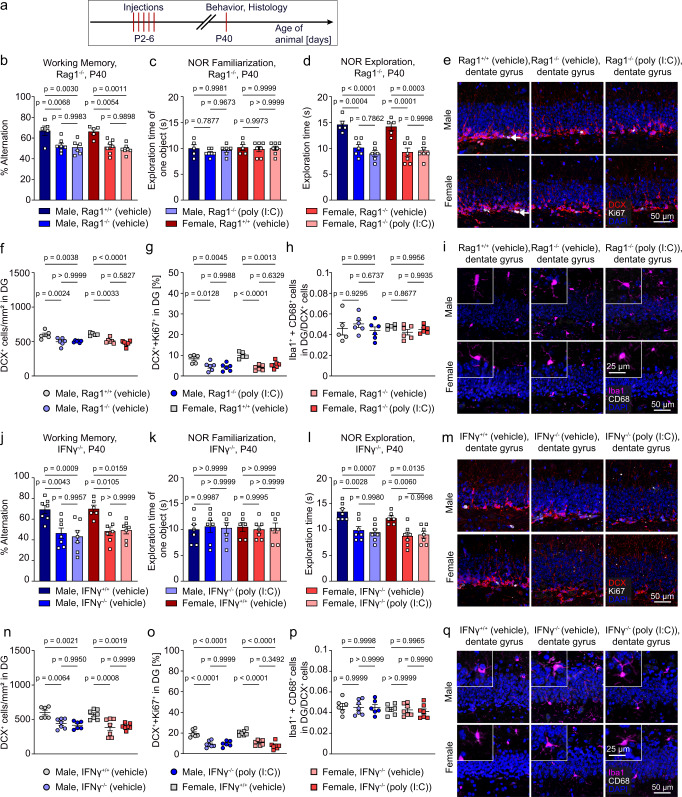
Behavioral deficits in male mice are induced by T cells and Interferon*-*γ. **a** Schematic diagram of the experimental timeline. **b** Percentage alteration in a T-maze test displayed by poly (I:C)-injected male and female *Rag1*^*−/−*^ mice compared to either vehicle-injected male and female *Rag1*^*−/−*^ mice or vehicle-injected Rag1^+/+^ mice of both sexes as shown in a bar graph at postnatal day 40. Data are presented as mean ± SEM. Each dot represents data of an individual mouse (*n* = 5 for Male, Rag1^+/+^ (vehicle); *n* = 7 for Male, Rag1^−/−^ (vehicle); *n* = 6 for Male, Rag1^−/−^ (poly (I:C)); *n* = 5 for Female, Rag1^+/+^ (vehicle); *n* = 7 for Female, Rag1^−/−^ (vehicle); *n* = 7 for Female, Rag1^−/−^ (poly (I:C))). Significant differences were determined by one-way ANOVA followed by Sidak multiple comparison test. *P* values are provided in the figure. **c** Exploration time in the novel object recognition test of one object during familiarization. Data are presented as mean ± SEM. Each dot represents data of an individual mouse (*n* = 5 for Male, Rag1^+/+^ (vehicle); *n* = 7 for Male, Rag1^−/−^ (vehicle); *n* = 6 for Male, Rag1^−/−^ (poly (I:C)); *n* = 5 for Female, Rag1^+/+^ (vehicle); *n* = 7 for Female, Rag1^−/−^ (vehicle); *n* = 7 for Female, Rag1^−/−^ (poly (I:C))). Significant differences were determined by one-way ANOVA followed by Sidak multiple comparison test. *P* values are provided in the figure. **d** Exploration time of a novel object in the novel object recognition test. Data are presented as mean ± SEM. Each dot represents data of an individual mouse (*n* = 5 for Male, Rag1^+/+^ (vehicle); *n* = 7 for Male, Rag1^−/−^ (vehicle); *n* = 6 for Male, Rag1^−/−^ (poly (I:C)); *n* = 5 for Female, Rag1^+/+^ (vehicle); *n* = 7 for Female, Rag1^−/−^ (vehicle); *n* = 7 for Female, Rag1^−/−^ (poly (I:C))). Significant differences were determined by one-way ANOVA followed by Sidak multiple comparison test. *P* values are provided in the figure. **e** Immunohistochemical detection at postnatal day 40 of Ki67 (white), DCX (red) and DAPI (blue) in the dentate gyrus of the hippocampus of poly (I:C)- or vehicle-injected *Rag1*^*−/−*^ and vehicle injected *Rag1*^*+/+*^ animals. Scale bar = 50 μm. Quantification of DCX^+^ cells is shown in figure (**f**). Quantification of DCX^+^+Ki67^+^ cells is shown in figure (**g**). Data are presented as mean ± SEM. Each color-coded symbol represents data of an individual mouse (*n* = 5 for Male, Rag1^+/+^ (vehicle); *n* = 6 for Male, Rag1^−/−^ (vehicle); *n* = 6 for Male, Rag1^−/−^ (poly (I:C)); *n* = 5 for Female, Rag1^+/+^ (vehicle); *n* = 6 for Female, Rag1^−/−^ (vehicle); *n* = 6 for Female, Rag1^−/−^ (poly (I:C))). Significant differences were determined by one-way ANOVA followed by Sidak multiple comparison test. *P* values are provided in the figure. **h** Quantitative analysis of Iba1^+^+CD68^+^ cells in direct contact with DCX^+^ cells in the dentate gyrus as shown in figure (**i**). Data are presented as mean ± SEM. Each color-coded symbol represents data of an individual mouse (*n* = 5 for Male, Rag1^+/+^ (vehicle); *n* = 6 for Male, Rag1^−/−^ (vehicle); *n* = 6 for Male, Rag1^−/−^ (poly (I:C)); *n* = 5 for Female, Rag1^+/+^ (vehicle); *n* = 6 for Female, Rag1^−/−^ (vehicle); *n* = 6 for Female, Rag1^−/−^ (poly (I:C))). Significant differences were determined by one-way ANOVA followed by Sidak multiple comparison test. *P* values are provided in the figure. **i** At postnatal day 40, brains were analysed by immunohistochemical labeling of CD68 (white), Iba1 (purple) and DAPI (blue) in the dentate gyrus of the hippocampus of poly (I:C)- or vehicle-injected *Rag1*^*−/−*^ and vehicle-injected *Rag1*^*+/+*^ animals. Scale bar = 50 μm. **j** Percentage alteration in a T-maze test of poly (I:C) injected male and female *IFNγ*^*−/−*^ mice compared to either vehicle injected male and female *IFNγ*^−*/−*^ mice or vehicle injected *IFNγ*^*+/+*^ mice of both sexes. Data are presented as mean ± SEM. Each dot represents data of an individual mouse (*n* = 7 for Male, IFNγ^+/+^ (vehicle); *n* = 7 for Male, IFNγ^−/−^ (vehicle); *n* = 7 for Male, IFNγ^−/−^ (poly (I:C)); *n* = 6 for Female, IFNγ^+/+^ (vehicle); *n* = 7 for Female, IFNγ^−/−^ (vehicle); *n* = 7 for Female, IFNγ^−/−^ (poly (I:C)). Significant differences were determined by one-way ANOVA followed by Sidak multiple comparison test. *P* values are provided in the figure. **k** Exploration time in the novel object recognition test of one object during familiarization. Data are presented as mean ± SEM. Each dot represents data of an individual mouse (*n* = 7 for Male, IFNγ^+/+^ (vehicle); *n* = 7 for Male, IFNγ^−/−^ (vehicle); *n* = 7 for Male, IFNγ^−/−^ (poly (I:C)); *n* = 6 for Female, IFNγ^+/+^ (vehicle); *n* = 7 for Female, IFNγ^−/−^ (vehicle); *n* = 7 for Female, IFNγ^−/−^ (poly (I:C)). Significant differences were determined by one-way ANOVA followed by Sidak multiple comparison test. *P* values are provided in the figure. **l** Exploration time of a novel object in the novel object recognition test. Data are presented as mean ± SEM. Each dot represents data of an individual mouse (*n* = 7 for Male, IFNγ^+/+^ (vehicle); *n* = 7 for Male, IFNγ^−/−^ (vehicle); *n* = 7 for Male, IFNγ^−/−^ (poly (I:C)); *n* = 6 for Female, IFNγ^+/+^ (vehicle); *n* = 7 for Female, IFNγ^−/−^ (vehicle); *n* = 7 for Female, IFNγ^−/−^ (poly (I:C)). Significant differences were determined by one-way ANOVA followed by Sidak multiple comparison test. *P* values are provided in the figure. **m** Immunohistochemical detection of Ki67 (white), DCX (red) and DAPI (blue) in the dentate gyrus of the hippocampus of poly (I:C)- or vehicle-injected *IFNγ*^*−/−*^ and vehicle-injected *IFNγ*^*+/+*^ animals was performed at postnatal day 40. Scale bar = 50 μm. Quantitative analysis of DCX^+^ cells is shown in figure (**n**). Quantification of DCX^+^+Ki67^+^ cells is shown in figure (**o**). Data are presented as mean ± SEM. Each color-coded symbol represents data of an individual mouse (*n* = 6 for Male, IFNγ^+/+^ (vehicle); *n* = 7 for Male, IFNγ^−/−^ (vehicle); *n* = 6 for Male, IFNγ^−/−^ (poly (I:C)); *n* = 7 for Female, IFNγ^+/+^ (vehicle); *n* = 7 for Female, IFNγ^−/−^ (vehicle); *n* = 7 for Female, IFNγ^−/−^ (poly (I:C)). Significant differences were determined by one-way ANOVA followed by Sidak multiple comparison test. *P* values are provided in the figure. **p** Quantification of Iba1^+^+CD68^+^ cells in direct contact with DCX^+^ cells in the dentate gyrus of the hippocampus of poly (I:C)- or vehicle-injected *IFNγ*^*−/−*^ and vehicle-injected *IFNγ*^*+/+*^ animals as depicted in (**q**). Data are presented as mean ± SEM. Each color-coded symbol represents data of an individual mouse (*n* = 6 for Male, IFNγ^+/+^ (vehicle); *n* = 7 for Male, IFNγ^−/−^ (vehicle); *n* = 6 for Male, IFNγ^−/−^ (poly (I:C)); *n* = 6 for Female, IFNγ^+/+^ (vehicle); *n* = 7 for Female, IFNγ^−/−^ (vehicle); *n* = 7 for Female, IFNγ^−/−^ (poly (I:C)). Significant differences were determined by one-way ANOVA followed by Sidak multiple comparison test. *P* values are provided in the figure. **q** Immunolabeling at postnatal day 40 for CD68 (white), Iba1 (purple) and DAPI (blue). Scale bar = 50 μm, 25 µm (insert). Source data are provided as a Source data file.

**Fig. 5:**
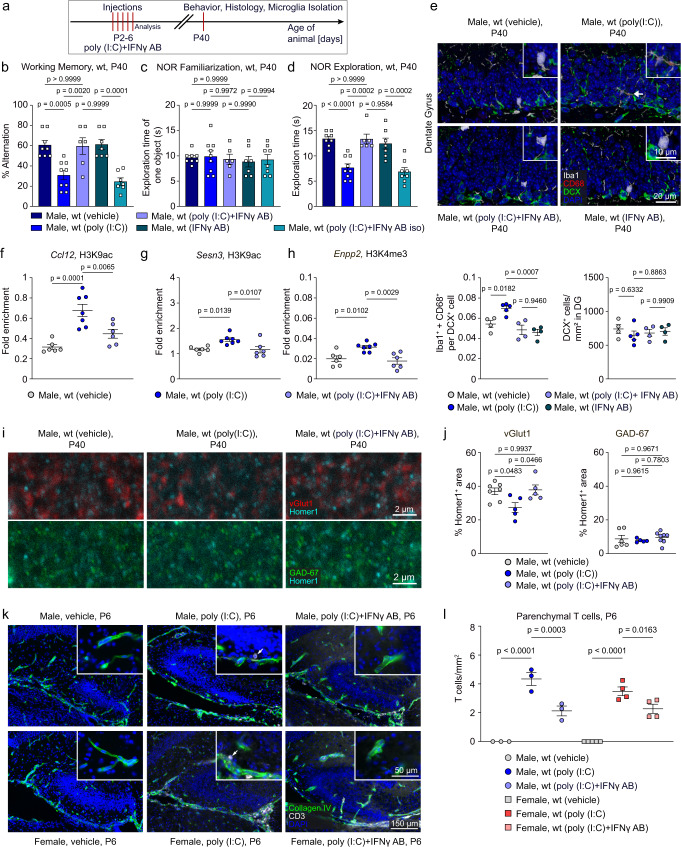
Interferon-γ neutralizing antibody prevents unfavorable effects induced by early poly (I:C) exposure. **a** The schema illustrates the individual steps of the workflow to obtain the data shown in Fig. 5b–q. **b** Percentage alteration in a T-maze test performed at postnatal day 40 shown by vehicle-injected male mice compared to male mice injected with poly (I:C), neutralizing IFNγ antibody, a combination of poly (I:C) and neutralizing IFNγ antibody or a combination of poly (I:C) + IFNγ antibody isotype control. Data are presented as mean ± SEM. Dots represent single mice (*n* = 8 for Male, wt (vehicle); *n* = 9 for Male, wt (poly (I:C)); *n* = 6 for Male, wt (poly (I:C) + IFNγ AB); *n* = 7 for Male, wt (IFNγ AB); *n* = 7 for Male, wt (poly (I:C) + IFN iso)). Significant differences were determined by one-way ANOVA followed by Sidak multiple comparison test. *P* values are provided in the figure. **c** Exploration time of one object in the novel object recognition test during familiarization. Data are presented as mean ± SEM. Dots represent single mice (*n* = 8 for Male, wt (vehicle); *n* = 9 for Male, wt (poly (I:C)); *n* = 6 for Male, wt (poly (I:C) + IFNγ AB); *n* = 7 for Male, wt (IFNγ AB); *n* = 8 for Male, wt (poly (I:C) + IFN iso)). Significant differences were determined by one-way ANOVA followed by Sidak multiple comparison test. *P* values are provided in the figure. **d** Exploration time of a novel object in the novel object recognition test. Identical treatment groups as employed for the T maze test (B) were used for the novel object recognition test (C,D). Data are presented as mean ± SEM. Dots represent single mice (*n* = 8 for Male, wt (vehicle); *n* = 9 for Male, wt (poly (I:C)); *n* = 6 for Male, wt (poly (I:C) + IFNγ AB); *n* = 7 for Male, wt (IFNγ AB); *n* = 8 for Male, wt (poly (I:C) + IFN iso)). Significant differences were determined by one-way ANOVA followed by Sidak multiple comparison test. *P* values are provided in the figure. **e** Immunohistochemical detection of CD68 (red), Iba1 (white), DCX (green) and DAPI (blue) in the dentate gyrus of male mice injected with vehicle, poly (I:C), poly (I:C) combined with neutralizing IFNγ antibody or injected with neutralizing IFNγ antibody alone (left) in P40 mice. Scale bar = 20 μm. Quantification of DCX^+^ cells and quantification of Iba1^+^+CD68^+^ cells in direct contact with DCX^+^ cells are shown below. Each color-coded symbol represents data of an individual mouse (*n* = 4 for Male, wt (vehicle); *n* = 5 for Male, wt (poly (I:C)); *n* = 4 for Male, wt (poly (I:C) + IFNγ AB); *n* = 4 for Male, wt (IFNγ AB)). Significant differences were determined by one-way ANOVA followed by Sidak multiple comparison test. *P* values are provided in the figure. **f**–**h** ChIP-qPCR analysis at P40 for H3K4me3 or H3K9ac occupancy on *Ccl12*, *Sesn3* and *Enpp2* promoters in microglia isolated from male mice, which were neonatally injected with vehicle, poly (I:C) or with poly (I:C) combined with neutralizing IFNγ antibody. Quantification of enrichment is represented as fold-enrichment over the background level. Single dots represent the data from one single mouse (*n* = 6 for Male, wt (vehicle); *n* = 7 for Male, wt (poly (I:C)); *n* = 6 for Male, wt (poly (I:C) + IFNγ AB)). Significant differences were determined by one-way ANOVA followed by Sidak multiple comparison test. *P* values are provided in the figure. **i** Positive immunofluorescent signals for Homer1 (turquoise), vGlut1 (red) and GAD-67 (green) in the dentate gyrus of the hippocampus of male mice at postnatal day 40 injected with vehicle, poly (I:C) and poly (I:C) combined with neutralizing IFNγ antibody. Scale bar = 2 µm. **j** Left panel: quantification of synapses by overlapping Homer1/vGlut1 positive signals. Data are presented as mean ± SEM. Single dots represent the data from one single mouse (*n* = 7 for Male, wt (vehicle); *n* = 5 for Male, wt (poly (I:C)); *n* = 5 for Male, wt (poly (I:C) + IFNγ AB)). Significant differences were determined by one-way ANOVA followed by Sidak multiple comparison test. *P* values are provided in the figure. Right panel: quantification of synapses by overlapping Homer1/GAD-67 positive signals. Data are presented as mean ± SEM. Single dots represent the data from one single mouse (*n* = 6 for Male, wt (vehicle); *n* = 5 for Male, wt (poly (I:C)); *n* = 8 for Male, wt (poly (I:C) + IFNγ AB)). Significant differences were determined by one-way ANOVA followed by Sidak multiple comparison test. *P* values are provided in the figure. **k** Immunofluorescence images of DAPI (blue), CD3 (white) and Collagen IV (green) from dentate gyrus of male and female neonatal mice (P6) injected with vehicle, poly (I:C) and poly (I:C) combined with neutralizing IFNγ antibody. Scale bares = 150 µm, 50 μm (insets). **l** Quantification of parenchymal CD3^+^ T cells on postnatal day 6. Pups were treated with vehicle, poly (I:C) and poly (I:C) combined with neutralizing IFNγ antibody. Data are presented as mean ± SEM. Each color-coded symbol represents one mouse (*n* = 3 for Male, wt (vehicle); *n* = 3 for Male, wt (poly (I:C)); *n* = 3 for Male, wt (poly (I:C) + IFNγ AB); *n* = 5 for Female, wt (vehicle); *n* = 4 for Female, wt (poly (I:C)); *n* = 4 for Female, wt (poly (I:C) + IFNγ AB)). Source data are provided as a Source data file.

We next determined whether behavioral deficits and the impact on DG immature neurons as seen in immune-challenged adolescent male mice were T cell-dependent by using recombination activating gene 1 (*Rag1*)^−/−^ mice. These mice have small lymphoid organs and do not develop mature T lymphocytes^38^. For the behavioral experiments, we mainly addressed spatial working memory and recognition memory. We omitted the three-chamber test for the study of social behavior because it has been previously reported that *Rag1*^*−/−*^ mice display impaired functional social recognition memory under homeostasis^39,40^. Early poly (I:C)-injection did not impair working memory (Fig. 4a, b) and recognition memory (Fig. 4c, d) in young adult male and female *Rag1*^*−/−*^ mice. For both sexes, immunohistological analyses of the dentate gyrus region revealed no effect of early poly (I:C)-injection on the number of immature neurons (Fig. 4e, f), on their proliferation rate (Fig. 4g), or on the number of activated microglia per DCX-positive neuron in *Rag1*^*−/−*^ mice when compared to *Rag1*^*+/+*^ littermates (Fig. 4h, i). It is apparent from these experiments that T cells represent a crucial catalyst for lasting behavioral and neuronal changes as a consequence of early immune stimulation in male animals. However, even under vehicle-treated conditions, male and female *Rag1*^*−/−*^ mice displayed mild memory deficits in both behavioral paradigms (Fig. 4a–d) as well as reduced neurogenesis (Fig. 4g) relative to *Rag1*^*+/+*^ littermates. We found that elevated forebrain IFNγ level as seen following poly (I:C) application in wt mice were abolished in *Rag1*^*−/−*^ mice of both sexes (Fig. S3d). To evaluate whether the increased IFNγ levels observed in the brains of poly (I:C)-treated male wt mice were responsible for the perturbed brain function in adolescence, we tested behavioral effects of neonatal poly (I:C) treatment in *IFNγ*^*−/−*^ mice. Similar to *Rag1*^*−/−*^ mice, vehicle-treated male and female *IFNγ*^*−/−*^ mice already displayed memory deficits (Fig. 4j–l) as well as defects in the neurogenic niche (Fig. 4m–o) relative to *IFNγ*^*+/+*^ control mice. However, in adolescent *IFNγ*^*−/−*^ mice of both sexes, the neonatal poly (I:C) treatment had no further detrimental effects on memory performance and adult neurogenesis when compared to vehicle-treated *IFNγ*^*−/−*^ mice (Fig. 4j–q). Since a decisive baseline level of IFNγ seemed essential for normal brain development already during embryonic development, we used a different approach and applied a purified anti-mouse IFNγ antibody (AB) to neutralize IFNγ following immune challenge in wild type mice (Fig. 5a)^41^. We only included male mice in this experiment since female mice did not show any late sequelae resulting from early immune stimulation. When neonatal male mice were co-injected with poly (I:C) and IFNγ AB, the memory impairment observed at P40, which was initiated by early poly (I:C) application, was prevented (Fig. 5b–d). In accordance with this finding, co-injection of IFNγ AB normalized the number of CD68-positive phagocytic active microglia that were in close contact to immature neurons (DCX^+^) found in the dentate gyrus (Fig. 5e). We then asked whether IFNγ AB co-treatment would interfere with poly(I:C)-induced epigenetic modifications in microglia. In order to address this question, we analyzed H3K4me3 and H3K9ac levels by ChIP-qPCR at the promoters of *Ccl12* (Fig. 5f), *Sesn3* (Fig. 5g) and *Enpp2* (Fig. 5h), which were differentially regulated in the RNA-seq data sets. In support of our behavioral and immunochistochemical data, we found neonatal co-application of IFNγ AB to reduce histone modifications, which were enhanced by poly (I:C) treatment (Fig. 5f–h). Application of poly (I:C) resulted in the preferential pruning of VGLUT1-positive synapses while GAD-67 positive synapses were not affected (Fig. 5i, j). Co-application of IFNγ AB prevented the pruning of excitatory synapses as seen after poly (I:C) was administered alone. The same IFNγ AB treatment reduced the number of T cells found in the brain parenchyma of male and female mice after poly (I:C)-exposure (Fig. 5k, l). To further strengthen our hypothesis that early IFNγ exposure drives later memory impairment we injected female mice directly with IFNγ during early postnatal stage and found memory impairment in both behavioral tasks when mice had reached adolescence (Fig. S4a–d). Early exposure of female mice to IFNγ also elicited an increase in H3K9ac at the *syntaxin 18* (*Stx18*) (Fig. S4e) and *Sesn3* (Fig. S4f) gene promoter region, respectively, while H3K4me3 levels at the *biogenesis of lysosomal organelles complex 1 subunit 5* (*Bloc1s5*) promoter region were not affected (Fig. S4g). *Stx18* is involved in endoplasmatic reticulum (ER)-mediated phagocytosis, where the ER fuses with the plasmalemma of macrophages underneath phagocytic cups^42^. *Bloc1s5* encodes a subunit of the BLOC1, a complex that is involved in the transport of some but not all cargo to the lysosome and lysosome-related organelles^43^. Although the early systemic application of IFNγ to female mice did not cause T cell invasion into the brain parenchyma (Fig. S4h), the spine numbers were reduced (Fig. S4i).

### Lasting adverse effects of early systemic MCMV infection in male mice

**Fig. 6:**
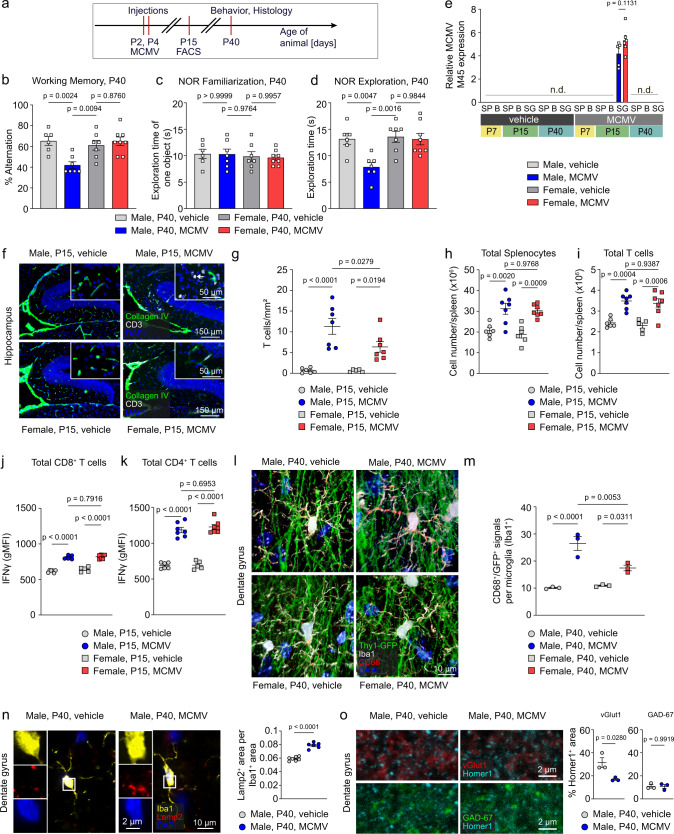
Neonatal MCMV infection causes synaptic and behavioral deficits in a sex-specific manner. **a** Schematic diagram of the experimental timeline. **b** Percentage alteration in a T-maze test displayed at postnatal day 40 by vehicle-injected mice compared to MCMV-infected mice of both sexes. Data are presented as mean ± SEM. Single dots represent the data from one single mouse (*n* = 6 for Male, P40, vehicle; *n* = 7 for Male, P40, MCMV; *n* = 7 for Female, P40, vehicle; *n* = 8 for Female, P40, MCMV). Significant differences were determined by one-way ANOVA followed by Sidak multiple comparison test. *P* values are provided in the figure. **c** Exploration time of one object in the novel object recognition test during familiarization at P40. Data are presented as mean ± SEM. Single dots represent the data from one single mouse (*n* = 6 for Male, P40, vehicle; *n* = 7 for Male, P40, MCMV; *n* = 7 for Female, P40, vehicle; *n* = 8 for Female, P40, MCMV). Significant differences were determined by one-way ANOVA followed by Sidak multiple comparison test. *P* values are provided in the figure. **d** Exploration time of a novel object in the novel object recognition test by vehicle-injected mice compared to MCMV-infected mice of both sexes. Data are presented as mean ± SEM. Single dots represent the data from one single mouse (*n* = 6 for Male, P40, vehicle; *n* = 7 for Male, P40, MCMV; *n* = 7 for Female, P40, vehicle; *n* = 8 for Female, P40, MCMV). Significant differences were determined by one-way ANOVA followed by Sidak multiple comparison test. *P* values are provided in the figure. **e** Viral genome equivalents relative to GAPDH in spleen (SP), brain (B) and salivary gland (SG) at postnatal days P7, P15 and P40 in vehicle- and poly (I:C)-injected mice of both sexes (*n* = 5–9 per age/sex/treatment combination) determined by quantitative RT-PCR. Data are presented as mean ± SEM. Single dots represent the data from one single mouse (*n* = 5 for Male, MCMV, P15, SG; *n* = 6 for Female, MCMV, P15, SG). Significant differences were determined by an unpaired, two-tailed *t*-test. The *p* value is provided in the figure (n.d.: not detectable). **f** Immunohistochemistry for CD3^+^ T cells (white), Collagen IV (green) and DAPI (blue) in the hippocampus of MCMV-infected or vehicle-injected animals obtained with P15 animals. Scale bars = 150 μm, 50 μm (insets). **g** Quantification of parenchymal CD3-positive T cells in hippocampal tissue at P15. Data are presented as mean ± SEM. Each color-coded symbol represents data obtained from one mouse (*n* = 6 for Male, P15, vehicle; *n* = 7 for Male, P15, MCMV; *n* = 5 for Female, P15, vehicle; *n* = 7 for Female, P15, MCMV). Significant differences were determined by one-way ANOVA followed by Sidak multiple comparison test. *P* values are provided in the figure. **h**, **i** Left panel: flow cytometry analysis and quantification of splenocytes isolated from spleen of male and female P15 mice, which were treated with vehicle or with MCMV. Data are presented as mean ± SEM. Each color-coded symbol represents data of an individual mouse (*n* = 6 for Male, P15, vehicle; *n* = 7 for Male, P15, MCMV; *n* = 6 for Female, P15, vehicle; *n* = 7 for Female, P15, MCMV). Significant differences were determined by one-way ANOVA followed by Sidak multiple comparison test. *P* values are provided in the figure. Right panel: flow cytometry analysis and quantification of T cells isolated from spleen of male and female P15 mice, which were treated with vehicle or with MCMV. Data are presented as mean ± SEM. Each color-coded symbol represents data of an individual mouse (*n* = 6 for Male, P15, vehicle; *n* = 7 for Male, P15, MCMV; *n* = 5 for Female, P15, vehicle; *n* = 7 for Female, P15, MCMV). Significant differences were determined by one-way ANOVA followed by Sidak multiple comparison test. *P* values are provided in the figure. **j**, **k** IFNγ production in CD8^+^ and CD4^+^ T cells (**k**) as measured by geometric mean fluorescence intensity (MFI) in P15 vehicle- and MCMV-injected mice of both sexes. Data are presented as mean ± SEM. Each color-coded symbol represents data of an individual mouse (*n* = 6 for Male, P15, vehicle; *n* = 7 for Male, P15, MCMV; *n* = 5 for Female, P15, vehicle; *n* = 7 for Female, P15, MCMV). Significant differences were determined by one-way ANOVA followed by Sidak multiple comparison test. P values are provided in the figure. **l** Immunohistochenistry in P40 mouse dentate gyrus of Iba1 (white), CD68 (red) and DAPI (blue) from vehicle- and MCMV-injected Thy1-GFP (green) male and female animals. Scale bar = 10. **m** Quantification of images shown under (l) by analysis of CD68^+^ + Thy1-GFP^+^ signals per microglia cell (Iba1^+^). Data are presented as mean ± SEM. Each color-coded symbol represents data of an individual mouse (*n* = 3 for Male, P40, vehicle; *n* = 3 for Male, P40, MCMV; *n* = 3 for Female, P40, vehicle; *n* = 3 for Female, P40, MCMV). Significant differences were determined by one-way ANOVA followed by Sidak multiple comparison test. P values are provided in the figure. **n** Immunohistochemical analysis of mice at P40 to detect Iba1 (yellow), Lamp2 (red) and DAPI (blue) in the dentate gyrus of the hippocampus of male vehicle-treated and MCMV-infected mice. Each color-coded symbol represents data of an individual mouse. Scale bars = 10 μm, 2 µm (insets). Quantification of Lamp2^+^ area per Iba1^+^ area is shown in the bar graph to the right. Data are presented as mean ± SEM. Each color-coded symbol represents data of an individual mouse (*n* = 7 for Male, P40, vehicle; *n* = 6 for Male, P40, MCMV). Significant differences were determined by one-way ANOVA followed by Sidak multiple comparison test. *P* values are provided in the figure. **o** Immunohistochemistry of Homer1 (turquoise), vGlut1 (red) and GAD-67 (green) in the dentate gyrus of the hippocampus of infected or vehicle-injected animals performed at postnatal day 40. Scale bar = 2 µm. Quantification of synapses is shown in the bar graph to the right. Each dot represents data of one individual mouse (*n* = 3 for Male, P40, vehicle; *n* = 3 for Male, P40, MCMV). Data are presented as mean ± SEM. Significant differences are determined by an unpaired two-tailed *t*-test. *P* values are provided in the figure. Source data are provided as a Source data file.

To evaluate whether the effects of a neonatal poly (I:C) challenge on adolescenct brain function could also be recapitulated by an actual viral infection, mice were peripherally infected with the mouse cytomegalovirus (MCMV) at days P2 and P4 (Fig. 6a). When mice were tested for cognitive performance at P40, MCMV-infected male mice showed poor working memory and reduced recognition memory, but cognitive function in MCMV-injected female animals could not be distinguished from control mice (Fig. 6b–d). We wanted to address the question whether MCMV was still present in mice at the time the behavioral experiments were performed since the virus impacts myeloid cell function^44^. Thus, we screened for the presence of viral DNA by PCR analysis of nucleic acid extracted from spleen (SP), brain (B) and salivary gland (SG). MCMV-specific DNA from the M45 gene coding region^45^ was only detected in the SG of males and females at P15 (Fig. 6e). The virus was not detectable at P7 or P40, or in tissue from vehicle-treated animals at any time points. Interestingly, although MCMV was never detected in the brain, the MCMV infection caused the infiltration of CD3^+^ T cells from the blood stream to the hippocampal brain parenchyma (Fig. 6f). Similar to what was observed following poly (I:C) treatment, more CD3^+^ T cells migrated into the brain of male than of female mice (Fig. 6g). To assess the immunological response to systemic MCMV infection, total splenocytes (Fig. 6h) and T cell populations (Fig. 6i) were analyzed by flow cytometry (Fig. S5). Male and female mice showed a similar increase in cell numbers. There were no distinctive, sex-specific differences in IFNγ production by CD8^+^ (Fig. 6j) and CD4^+^ T cells (Fig. 6k) 11 days after the MCMV infection. In order to investigate whether sexual dimorphism in microglial response was also present after early MCMV infection, we measured microglial phagocytic activity in the hippocampus of male and female mice at P40 (Fig. 6l, m). We performed immunocolocalization analysis of hippocampal neurons in the DG region (Thy1-GFP) with microglial cytoplasm (Iba1), which shows positive signals for the phagosomal marker CD68. Our analysis revealed that microglia colocalization was higher in males than in females (Fig. 6m). In further support of these findings, the strong phagocytic activity of microglia in MCMV-infected mice was highlighted by large cytosolic Lamp2^+^ areas, which denote regions of phagocytic activity^46^ (Fig. 6n). It was recently shown that microglia were contributing to a late phase refinement by transiently engulfing and removing synapses between P30 and P60^47^. In early MCMV-infected mice, there was a significant reduction in the density of excitatory synapses (vGlut1^+^/Homer1^+^) while inhibitory synapses (GAD67^+^/Homer1^+^) were not affected (Fig. 6o).

## Discussion

The results of our present study link early peripheral immune stimulation to behavioral changes later in life. In response to neonatal poly (I:C)- or MCMV-injections, IFNγ level increased in the periphery and in the brain and T cells entered the brain parenchyma. IFNγ led to an epigenetic programming of microglial cells, as indicated by increased H3K9 acetylation and H3K4 trimethylation, that lasted until at least adolescence specifically in male mice. As a result, microglial phagocytosis remained highly activated and maintained a compromised neural network in the hippocampal dentate gyrus. The immediate effects of this disturbed neural network development were hippocampus-dependent behavioral deficits, which affected working memory, recognition memory and social behavior. These behavioral impairments were exclusively seen in male mice and absent in female mice.

In previous studies, it was already suggested that early immune stimulation and long-term behavioral deficits may be closely intertwined. However, here it is important to distinguish between pre- and postnatal immune activation. Maternal immune activation (MIA) has become a well-established disease model for studying schizophrenia and ASD^48–50^. During pregnancy, maternal exposure to bacterial (lipopolysaccharide, LPS) or viral (poly (I:C)) mimetics causes alterations in the offspringʼs brain development and function, as well as in their behavior^51^. Similar to our findings employing neonatal immune stimulation, a poly (I:C)-induced prenatal immune challenge at E9.5 triggers behavioral deficits and microglial changes preferentially in males^52^.

While prenatal immune stimulation (e.g., MIA) has been thoroughly examined over the last years, relatively little is known about the impact of an early postnatal challenge on CNS function. There is one study, for instance, that found sex-specific memory impairments following stimulation with LPS or poly (I:C)^53^. Early immune stimulation by postnatal LPS administration led to a long-term increase in spontaneous epileptiform activity^54^. Furthermore, it has been shown that a subcutaneous administration of LPS at postnatal day 9 induces alterations in feedforward GABAergic inhibitory postsynaptic responses^55^. In general, LPS was shown to induce elevated cytokine levels in both serum and the brain, as well as sickness- and depressive-like behavior in male mice^56^. Decreased social behavior is a hallmark aspect of sickness behavior. Another study, which found lower sociability in adult female mice but not male mice following early administration of LPS, rules out cytokines as a probable reason and establishes a link between increased somatostatin interneurons in the anterior cingulate cortex and social behavioral abnormalities^57^. Despite all of this data, it still unclear what is to blame for behavioral impairments in adult male mice after early immune activation. It is also uncertain why female mice seem to be protected from the adverse effects seen later in life even though they have experienced the same early immune challenge as male mice. While our results stress the importance of IFNγ as a key player for the epigenetic programming of microglial cells, the response to IFNγ does not seem to be hampered in female mice. Injection of IFNγ elicited similar detrimental behavioral effects in combination with epigenetic modifications as seen in male mice after early poly (I:C) or virus exposure.

After postnatal poly (I:C) injections we observed significantly higher IFNγ levels in the spleen of male compared to female mice, which correlated with higher *IFNγ* mRNA expression levels. This finding might be explained by differences in gonadal steroids. However, the specific role of estrogen (17β-estradiol) in modulating IFNγ levels is still obscure because estrogenic compounds have been reported to either suppress or enhance IFNγ production in splenocytes^58,59^. Allthough in our experiments the immune challenge occurred during the neonatal phase where gonadal steroids are low^60^, the neonatal sex steroid milieu might still be sufficient to affect the female IFNγ response. Previous research has shown that there are sex variations in susceptibility to a viral infection, which are accompanied by a lower CD4^+^ T cell mediated response, including expression of IFNγ in female mice. To a certain extent this finding was attributed to decreased signaling of TLR9 in female than male mice^61^. However, in our experimental settings poly (I:C) injections did not cause any downregulation of the *Toll-like receptor-3* (*Tlr3*) mRNA in the spleen of female mice. This observation excludes the possibility that different IFNγ level are caused by sex-dependent changes in TLR3 expression. Several studies demonstrated that IFNγ promotes the transendothelial migration of T cells into the central nervous system^62,63^. Higher IFNγ levels in the periphery may explain the potentially higher migration of T cells in male mice. However, our data obtained after MCMV-infection argue against this hypothesis. After a systemic MCMV infection, the number of T cells in the periphery was identical between male and female mice. Importantly, similar to the situation after poly (I:C) injections, the number of T cells in the brain parenchyma was higher in males than in females. The finding that systemic IFNγ in females induced identical effects as seen after poly (I:C) application in males without an increase in parenchymal T cells shows that robust levels of IFNγ generated in the periphery can initiate modified CNS function in females. Specific features of the the blood-brain barrier (BBB) might contribute to higher numbers of parenchymal T cells but also to higher IFNγ levels in the male brain. There might be differences in the available mechanisms necessary for T cell migration and IFNγ transport across the BBB between male and female mice due to variable stages of development. Furthermore, IFNγ may have very deviating modulatory effects on the cerebral vasculature in male and female mice, which results in dysregulation or deterioration of BBB integrity^55^. The BBB might also differ in maturation in both sexes. Thus, early exposure to an immune challenge may favor transmigration of T cells and the IFNγ transport from the periphery to the brain in male mice^64^. Notably, the effects including behavioral deficits were absent in male poly (I:C)-injected *IFNγ*^*−/−*^ mice and in male poly (I:C)-injected *Rag1*^*−/−*^ mice, which lack mature B and T cells. Since B cells are not IFNγ producers, IFNγ originating from T cells was indeed the main source for lasting behavioral and neuronal changes. Since untreated *Rag1*^*−/−*^ and *IFNγ*^*−/−*^ mice showed neurogenesis and memory deficits, IFNγ baseline levels may be required for normal brain development and function^65^. The fact that poly (I:C) did not impair the cognitive performance or adult neurogenesis in *Rag1*^*−/−*^ and *IFNγ*^*−/−*^ mice any further suggested a key role for T cells and IFNγ in these processes, which were not further intensified by poly (I:C) because a so-called ceiling effect had most likely been reached. In order to avoid the baseline effects seen in *IFNγ*^*−/−*^ mice, we changed the mouse model and systemically administered a neutralizing IFNγ antibody to wild type mice. When the antibody was co-injected with poly (I:C), the cognitive impairment seen after poly (I:C) injection alone was no longer present. In the case that the antibody is unable to cross the blood-brain-barrier, the results would then suggest a contribution of peripheral IFNγ to this process. Indeed, a recent study described a critical role for meningeal IFNγ in mediating both neural function and social behavior^66^. IFNγ was produced by meningeal T cells, which never showed any transmigration. This discrepancy to our findings is most likely based on the very different ages of mice used in both studies, which seems to be critical for T cell transmigration. We were stimulating mice as neonates, Filiano et al. used adult mice^66^. Even though the effects of IFNγ are far from being completely understood, there is emerging evidence that the cytokine is involved in chromatin-modifying changes that could regulate the expression of other genes. In particular, it has been shown that IFNγ modulates the mRNA expression of epigenetic regulators in human blood monocyte-derived macrophages^67^. Moreover, IFNγ is capable of priming and inducing de novo enhancer formation to promote activation of gene transcription^68^. In our present work, early immune activation resulted in epigenetically-modified microglia, which is in accordance with previous data from our and other groups indicating that acetylation and methylation of H3 are associated with altered microglial function after an immune challenge^69,70^. It might seem surprising that *Enpp2* expression was downregulated in males after poly (I:C) challenge while levels of H3K4me3 were increased because H3K4me3 is in general believed to facilitate gene expression^71^. However, the fact that lack of H3K4me3 has no major impact on the transcription of numerous genes and the observation that transcription of some genes can be negatively or positively regulated, argues against its general role as an activator of gene transcription^71^. The fact that H3K4me3 may attract a number of different factors altering transcription, can explain why H3K4me3 might have both, activating or repressing function.

Particularly in male mice, epigenetic changes occurred together with prolonged microglial activation, which caused excessive pruning of excitatory synapses in the adult hippocampal neuronal network. This microglia-typical extensive surveillance of the brain parenchyma to fine-tune neural circuits spared inhibitory synapses. How microglia-synapse interactions differ between excitatory and inhibitory synapses is still unclear, but in our hands, using a perinatal infection model, the enhanced neuron pruning by microglia should result in a lower overall activation status of the brain. Our data reveal that microglia’s modulatory effects can last long after the pathogenic process has been terminated. One far-reaching question of these lasting alterations in neuroimmune function is whether the brain has the ability to fully reestablish homeostasis. If not, early sickness like infections that activate the immune system may cause not only the initial activated immune state, but also a new, permanently altered state of the male brain. Females appear to have a greater intrinsic threshold to endure the far-reaching influence of early immunological assaults on brain function, either totally or partially.

## Methods

### Mice

Animal studies were approved by the Regional Council of Freiburg, Germany, and the Research Ethics Committee at Leuven University, Leuven, Belgium. Male and female mice were used for the experiments. Mice were group housed up to five per cage with 12 h light/dark cycle with lights on at 6 a.m. Food and water were available *ad libitum*. Ambient temperature was 20–22 °C with 40–60% humidity. C57BL/6 J mice were used as WT mice. *Rag1*^*−/−*^ mice (strain no. 002216), *IFNγ*^*−/−*^ mice (strain no. 002287) and Thy1-GFP-M transgenic animals (strain no. 007788) all on C57BL/6 J genetic background, were obtained from the Jackson Laboratories. Tissue obtained from a female 8 week old *Cx3cr1*^*cre*^ *x Usp18*^*fl/fl*^ mouse^72^ served as a positive control for western blots.

### Intraperitoneal injections

Neonatal mice were injected intraperitoneally (i.p.) with poly (I:C) [5 mg/kg] or vehicle once daily from postnatal day 2 to postnatal day 6. WT bacterial artificial chromosome (BAC) derived, MCK-2 repaired MCMV^73^ (60 PFU^44^) was also given i.p. at postnatal days 2 and 4. To deplete IFNγ, we coinjected 0.5 mg of IFNγ monoclonal antibody (mAb) (XMG1.2; Leinco). IFNγ was injected at P2, P4, and P6 at a concentration of 10^4^ IU/kg.

### Behavioral testing

#### Three-chamber social preference test

Sociability and social novelty was assessed in a three-chamber social approach apparatus using a modification of the previously described protocol^74^. Briefly, mice were placed in a rectangular apparatus divided into 3 chambers by transparent partitions with small circular openings allowing easy access to all compartments. In a 10-min session, an age- and sex-matched, unfamiliar C57BL/6 J mouse (M) was placed in one of the two wire cages. The wire cage on the other side remained empty (E). The test mouse was placed in the center, and allowed to freely explore the chamber. In a social novelty test session (10 min), an age- and sex-matched C57BL/6 J stranger mouse (M2) was placed in one wire cage, the other wire cage was occupied by an age- and sex-matched, familiar C57BL/6 J mouse (M1). Thus, the test mouse had the choice to interact with a mouse that was already familiar (M1) or with a new stranger mouse (M2). The movement of the mouse was recorded by a video camera and PC-based video capture software. The recorded video file was further analyzed by off-line video tracking software (BIOBSERVE, Germany). Time spent in each chamber was measured.

#### T-maze test

Spatial working memory was tested using the T-maze test as previously described^46^. Training of a mouse consisted of one single session, which started with one forced-choice trial, followed by 14 free-choice trials.

#### Novel object recognition test

The Novel Object Recognition (NOR) task is used to evaluate cognition, particularly recognition memory. During habituation, the animals were allowed to explore an empty arena for 10 min. Twenty-four hours after habituation, the animals were exposed to the familiar arena with two identical objects placed at an equal distance. After 6 h, the mice were allowed to explore the open field in the presence of the familiar object and a novel object to test long-term recognition memory. The time spent exploring each object was recorded. The experiment was stopped for each run when mice had interacted with both objects for a total time of 20 s.

### Ex vivo isolation of microglia and splenocytes and flow cytometry

Mice were anesthetized and transcardially perfused with ice cold PBS. Brain hemispheres were homogenized and subjected to 37% percoll density gradient separation. Microglia cells were collected from the interface of 37% percoll and washed extensively with FACS buffer (0.5% FBS in PBS) followed by staining with anti-CD11b (clone M1/70, eBioscience, 1:200) and anti-CD45 (clone 30-F11, eBioscience, 1:200) antibodies. Isolation of immune cells and flow cytometry. Splenocytes were obtained by gentle pressure-dissociation of spleen in HBSS supplemented with 1% HEPES buffer (pH 7) and glucose, and then passed through a sterile 100 μm cell strainer. Red blood cells were lysed using 3 ml of RBC lysis buffer (Bio-Legend, San Diego, CA) and incubated for 5 min on ice. The reaction was stopped by adding 8 mL of ice-cold FACS buffer. Splenocytes were counted on a hemocytometer and resuspended in DMEM supplemented with 10% FBS and L-glutamine to a density of 2 × 10^6^ cells/ml. Splenocytes were then stimulated with PMA (5 ng/ml, Sigma) and ionomycin (1 μM; Sigma) in the presence of brefeldin A (5 μg/ml; BioLegend) for 3 h at 37 °C. After the incubation, splenocytes were stained with fixable viability dye (eF780, eBioscience) for 20 min, blocked using Fc block for 5 min (clone 2.4G2, BD Biosciences), and stained for cell surface antigens for 30 min. Then, the cells were sequentially fixed and permeabilized using Fixation Buffer and Intracellular Staining Permeabilization Wash Buffer (both from BioLegend), and stained for intracellular antigens. All steps were performed on ice and the antibodies used were CD3ϵ (clone 145-2C11, eBioscience, 1:200), CD4 (clone RM4-5, BD Biosciences, 1:200), CD8a (clone 53-6.7, eBioscience, 1:200), CD49d (clone R1-2, BioLegend, 1:200), CD11a (clone M17/4, eBioscience, 1:200), CD19 (clone 1D3, BD Biosciences), NK1.1 (clone PK136, Biolegend) and IFNγ (clone XMG1.1, eBioscience, 1:200). Cells were sorted using a FACSAria III (Becton Dickinson) and used for further analysis.

### RT-qPCR and RNA sequencing

RNA from FACS-purified microglia was purified using the Arcturus PicoPure RNA Isolation Kit (ThermoFisher Scientific) and cDNA was prepared using the High-capacity RNA-to-cDNA Kit (Applied Biosystems), both according to the manufacturer’s recommend protocol. Gene expression was quantified by RT-qPCR using the LightCycler480 SYBR Green I Master (Roche) on a LightCycler480 instrument (Roche), and analyzed by the ΔΔCt method using *Gadph* as a reference gene. Primers used for RT-qPCR can be found in Table 1. After MCMV-infection organs were harvested at P7, P15 and P40 and 1 µg of isolated gDNA was used to perform RT-qPCR. Viral M45 was normalized to cellular GAPDH. RNA-Seq was performed and analyzed as previously described^46^.

**Table 1:** Primers used for RT-qPCR experiments

Target	Forward primer	Reverse primer	Annealing temperature
*Gapdh*	TCCTGCACCACCAACTGCTTAGCC	GTTCAGCTCTGGGATGACCTTGCC	Individual
*Ccl2*	TCTGGGCCTGCTGTTCAC	TTGGGATCATCTTGCTGGTG	64 °C
*Ccl12*	GCTACCACCATCAGTCCTCAGG	CTTCCGGACGTGAATCTTCTG	60 °C
*Cxcl10*	TGCTGGGTCTGAGTGGGACT	CCCTATGGCCCTCATTCTCAC	64 °C
*Cxcl11*	CCACAGCTGCTCAAGGCTTCCT	GCGAGCTTGCTTGGATCTGGGG	64 °C
*Ifi204*	TGCTACCGTGGCTACTGAAA	GCCACCCATCTTCAGTGGAA	60 °C
*Tnfα*	TCTTCTCATTCCTGCTTGTGG	AGGGTCTGGGCCATAGAACT	60 °C
*IFNβ*	CTCCACCACAGCCCTCTCCA	TCTGCATCTTCTCCGTCATCTCC	64 °C
*IFNγ*	AGGAACTGGCAAAAGGATGGT	TCATTGAATGCTTGGCGCTG	62 °C
*Enpp2*	GCTGCACCTGTGATGATAAGG	GCAGGTCGTCCATACAGGAG	60 °C
*Jun*	TGAGTGACCGCGACTTTTCA	GAGGGCATCGTCGTAGAAGG	60 °C
*Sesn3*	ACTACCTGCTCTGCACCAAC	GTCAGGGGTTGAGACACTCG	60 °C
*Tlr3*	TTGCGTTGCGAAGTGAAGAA	TCGAGCTGGGTGAGATTTGTCC	62 °C
*M45*	ATCTCCTCGAAGGGGAATGA	TCGACAGACAGCCGTTCGT	60 °C
*Gapdh (genomic)*	CTGCAGTACTGTGGGGAGGT	CAAAGGCGGAGTTACCAGAG	60 °C

### RNAseq data analysis

Quality of sequencing reads stored in FASTQ files was assessed using FastQC (v0.67) and trimmed using Trim Galore! (v0.4.3). Reads were mapped on the mouse genome version mm10 (UCSC) using STAR aligner (v2.5.2)^75^ with RefGene annotation. The number of reads mapped to each gene (counts) was extracted from the BAM files using FeatureCount (v1.5.3)^76^ with the annotation version mm10 from UCSC and the following parameters: exon feature file, unstranded, a minimum mapping quality per read of 12, a minimum overlap of 1 bp and other parameters set to default. A quality report of each step was generated using MultiQC (v1.5.0)^77^. R (v3.4.3) was used to perform the Ward error sum of squares hierarchical clustering method^78^ and principal components analyses (PCA). The process to extract the gene counts from FASTQ files was run on Galaxy^79^. Using the DESeq2 model, the differentially expressed genes (DEGs) showing adjusted *p*-values (Wald test) of less than 0.05 and log 2 fold change greater than 0.97 were identified. Heatmaps were plotted using the pheatmap R package (v1.0.8) calculated from scaled (Z-scores), normalized read counts of DEGs with a hierarchical clustering of the rows. Normalized counts generated by DESeq2 were assessed for artifacts or contamination by other cell types. The list of genes used was based on single-cell RNA-sequencing data from the mouse Allen Brain Map cell types database^80^.

### Chromatin immunoprecipitation (ChIP)

Brain single cell suspensions were cross-linked in 1% PFA for 5 min, and PFA was quenched with 0.25 M glycine for additional 5 min. Microglia were then purified by FACS-sorting as described above. Following sorting, purified microglia were lysed in 400 μL of lysis buffer (0.2% NP40, 10 mM Tris-HCl pH 8, 10 mM NaCl containing protease inhibitors (Roche cOmplete™ Protease Inhibitor Cocktail Tablets) and 10 mM butyrate) for 10 min. Nuclei were resuspend in 130 μL of shearing buffer (0.1% SDS, 10 mM Tris-HCl pH 8, 1 mM EDTA, protease inhibitor cocktail, 10 mM butyrate) and the DNA was sheared using the Covaris M220 Focused-ultrasonicator (PIP 75 W, dr 5%, 200 cycles/burst). Immunoprecipitation was performed on the sheared DNA from 50,000 microglia using the immunoprecipitation buffers from the iDeal ChIPseq Kit for Histones (Diagenode). Briefly, sheared DNA was incubated with 0.5 μg of antibody overnight (rotating, 4 °C) in 300 μL of ChIP buffer. The next day, 10 μL of protein A magnetic beads (Diagenode) was added to the reaction and incubated for 3 h (rotating, 4 °C). The beads were then washed with each wash buffer once for 5 min each, and the bead-antibody-histone-DNA complexes were dissociated with 100 μL of Elution Buffer for 30 min at room temperature. Samples were decrosslinked with 0.2 mg/mL of proteinase K and 0.1 mg/mL of RNase A first at 37 °C for 1 h, then at 65 °C overnight. DNA was cleaned up using Minelute PCR Purification Kit (Qiagen) according to the manufacturer’s instructions. ChIP enrichment was tested by qPCR using primers against the promoter of *Gapdh* (positive control) and a gene desert (Des1, negative control) and compared to an input control. All primers used for ChIP-qPCR experiments can be found in Table 2, and were designed against high H3K4me3 binding regions found within 1 kbp of the respective gene promoter^81^ using the UCSC Genome Browser tool. Antibodies used for ChIP were ChIP-grade H3K4me3 (abcam, ab8580) and H3K9ac (abcam, ab10812).

**Table 2:** Primers used for ChIP-qPCR experiments

Target	Forward primer	Reverse primer
*Gapdh*	CTCCTGGCTTCTGTCTTTGG	TGGCGTAGCAATCTCCTTTT
*Bloc1s5*	TGCCAGGAAGAACACTTGG	TCGAAATCTACTAAGGCTGAAAGG
*Ccl12*	ACTTCCTATTGCTGGCCTCA	CTCAGCCAGAAGGAAGCTGT
*Enpp2*	CGATGGCAAAAGTGAACAAG	TCTGAACTGCTTCCATGCAC
*Sesn3*	GACCCCCTGAGAAGAGAACG	TGGTGCAGAGCAGGTAGTTG
*Stx18*	CCAAACTGGGCCTATCTCTTTAG	CGTCCCTTCGTGAGAATAAACC

All primers were used with an annealing temperature of 60 °C.

### Organ homogenates and IFNγ quantification

At day 6 (3, 7, 12, 24, 36 h.p.i.) the spleens and forebrains (meninges were removed) were harvested and homogenized in 500 µl PBS with phenylmethylsulfonil fluoride (PMSF; Sigma, St Louis, USA) using a tissue homogenizer. Further homogenization was performed using the Sonifier Cell Distuptor B15 (Branson Ultrasonics, Danbury, USA). The samples were centrifuged at 10,000 g for 25 min at 4 °C. Supernatants were collected and stored at −80 °C until analysis by sandwich enzyme-linked immunosorbent assay (ELISA). Optical densities were measured at 450 nm, using a microplate ELISA reader (Biorad, Hercules, USA).

### Western blot

Neonatal microglia were isolated from P6-8 male and female mice using the same procedure as for adult microglia. Following Percoll gradient density centrifugation, cells from the same sex were pooled (up to 5 brains) and MACS-enriched using CD11b-biotinylated (clone M1/70, eBioscience) and MACS anti-biotin Microbeads (Miltenyi Biotec) following the manufactures recommended protocol. Enriched microglia (200,000 cells per condition) were stimulated with recombinant mouse IFNγ (rmIFNγ) as indicated in the figure legends. Cell lysates were prepared in 20 μL of RIPA buffer containing cOmplete Protease Inhibitor (Roche) and sodium orthovanadate on ice for 30 min. Lysates were run on a 10% SDS-PAGE gel and blotted for pSTAT1(Y701) (Cell Signaling Technology) and β-actin-HRP (Cell Signaling Technology). pSTAT1(Y701) bands were quantified using ImageJ and normalized to the respective β-actin band. Spleen homogenate from *Cx3cr1*^*cre*^ *x Usp18*^*fl/fl*^ adult mice treated intraperitoneally with IFNβ for 24 h was used as a positive control for pSTAT1(Y701)^72^.

### Fluorescence microscopy

After transcardial perfusion with phosphate-buffered saline (PBS), brains were fixed in 4% PFA and embedded. 14-μm cryosections were blocked with PBS containing 5% bovine serum albumin and permeabilized with 0.1% Triton X-100 in blocking solution. The following primary antibodies were added overnight at 4 °C to label: Iba‐1 for microglia (1:500, WAKO, Japan), DCX for immature neurons (1:250, Santa Cruz Biotechnology, USA), Ki67 (1:100, Invitrogen, USA) to detect proliferating cells, CD68 (1:100, BioRad, UK) to detect macrophages/activated microglia and cleaved caspase-3 (1:100, Cell Signaling Technology, USA), which is involved in the activation cascade of caspases responsible for apoptosis execution. The lysosome was labeled with an antibody against Lamp2 (1:200, Abcam, UK), the post synaptic density (PSD) protein Homer1 was detected by antibodies against Homer1 (1:500, Millipore, USA), glutamatergic neurons by anti-vGlut1 antibodies (1:200, Synaptic Systems, Germany) and GABAergic neurons were detected by anti-GAD67 antibodies (1:500, Sigma, USA). Primary antibodies against CD3 (1:100, BioRad, USA) detected T cells, anti-collagen IV antibodies (1:250, Millipore, USA) labeled blood vessels. Secondary antibodies were added as follows: Alexa Fluor 488, 1:500; Alexa Fluor 647, 1:500; Alexa Fluor 568, 1:500, for 2 h at room temperature.

### Quantitative analysis

For all quantifications, counting was performed manually by a blinded observer using the original images. Reconstruction was carried out in order to allow for visualization. For Figs. 2a, 4f–i, 4n–q, 5e and Supplementary Fig. 2a, b, DCX-immunoreactive cells in the DG were counted in a 1 in 10 series of sections throughout the whole hippocampus. For the quantification of DCX‐, Iba-1-, Ki67-, and CD68- positive cells, alone or in combination, representative images were obtained by using a Keyence BZ-9000 Biorevo microscope. For Fig. 6n Lamp2-positive areas per Iba-1-positive microglia were determined using ImageJ. Parenchymal CD3-positive T cells that are characterized by an extravascular location outside collagen-IV-positive structures were quantified on three sections per hippocampus (Figs. 3e; 6f, g). For Figs. 2e and 6l images were taken using an Olympus Fluoview 1000 confocal laser scanning microscope equipped with a 20 × 0.95 NA objective. For z-stacks a step size of 0.6 µm was chosen and 3D images with a resolution of 1024 × 1024 pixel were analyzed using IMARIS software, version 9.6.0 (Bitplane). To determine the number of CD68- and GFP-double positive signals per microglia cell (Iba-1), we manually quantified the number of contact points, which displayed positive signals for all three antibody markers.

### Quantification of pre- and post-synaptic marker proteins

The sections (7 µm) were scanned with an Olympus Fluoview 1000 confocal laser scanning microscope using a 63 × 1.4 NA objective and 4x zoom. The resulting z-stacks with 0.1-μm steps in z direction and 1024 × 1024 pixel resolution were analyzed using IMARIS software (Bitplane). To analyze the association of Homer1 puncta with glutamatergic or GABAergic boutons, which was defined as synapse, we quantified the proportion of boutons which were in direct contact with at least one Homer1 punctum by following the bouton through its rostrocaudal length in the confocal z-series (Figs. 5i, j and 6o).

### Spine image acquisition and quantification

For quantification of dendritic spines (Fig. 2b–d; S4I), images were captured on an Olympus Fluoview 1000 confocal microscope with a 63 × 1.4 NA objective and z-series (z-step of 0.1 µm, a system optimized value which offered the best z-resolution and kept the amount of bleaching to a minimum) with 4x zoom and 1024 × 1024 pixel resolution. Images were analysed using IMARIS software (Bitplane). To assess spine density, a minimum of 5–7 dendritic segments (length 10–30 µm) for each mouse brain was randomly selected and quantified.

### Three-dimensional reconstruction

30 μm coronal cryo-sections from brain tissue were stained with anti-Iba-1, anti-DCX and anti-CD68 (same concentrations as above) for 48 h followed by AlexaFluor-conjugated secondary antibodies, which were added for additional 24 h at 4 °C. Imaging was performed on an Olympus Fluoview 1000 confocal laser scanning microscope (Olympus) using a 20 × 0.95 NA objective. Z-stacks were obtained with 0.8-μm steps in z direction and 1024 × 1024 pixel resolution (Fig. 2a). Dendrites and dendritic spines were reconstructed from Thy1-GFP transgenic mice (Fig. 2b–d). Imaging was performed on an Olympus Fluoview 1000 confocal laser scanning microscope (Olympus) using a 63 × 1.4 NA objective with a 4x zoom. Z-stacks were obtained with 0.1-μm steps in z direction and 1024 × 1024 pixel resolution. Reconstruction was performed using IMARIS software (Bitplane).

### Chromogenic immunohistochemistry

Images of chromogenic DAB immunohistochemistry of CD3-positive T cells were acquired using a BZ-9000 Keyence microscope (Biorevo) equipped with a brightfield filter (Fig. S4h). Chromogenic immunohistochemistry of 3 µm thick FFPE sections was perfomed as previously described^82^. The primary antibody against CD3 was obtained from BioRad (1:100). Goat Anti-Rat IgG (1:200, SouthernBiotech) served as secondary antibody antibody. Images were acquired using a BZ-9000 Keyence microscope (Biorevo) equipped with a brightfield filter.

### Statistics and reproducibility

To obtain unbiased data, experimental mice of all relevant genotypes were processed together and cell quantifications were carried out blinded to the genotype. Only after finalization of all quantitative measurements were the samples allocated to their genotypes. In all behavioral assays, subjects were randomly assigned to a group and the experiments were blind with respect to group assignments. No statistical methods were used to predetermine sample sizes and exact group numbers were determined by animal availability. However, we did ensure that our sample sizes were similar to those generally employed in the field and all experiments were replicated at least once^83–85^. No data or mice (n) were excluded. If not indicated otherwise, all experiments were performed once. Statistical analysis was performed using GraphPad Prism (GraphPad Software, Version 9.5.1). For comparisons between multiple groups, ANOVA was used followed by Sidak *post hoc* test when applicable. When two groups were compared, an unpaired two-tailed *t*-test was applied. Unless indicated otherwise, data are expressed as mean ± SEM.

### Reporting summary

Further information on research design is available in the Nature Portfolio Reporting Summary linked to this article.

## Supplementary information

Supplementary Information

Description of Additional Supplementary Files

Supplementary Data 1

Supplementary Data 2

Reporting Summary

## Data Availability

Source data are provided with this paper. Bulk RNA sequencing datasets were deposited into the Gene Expression Omnibus database under accession number GSE198473 and are available at the following URL: https://www.ncbi.nlm.nih.gov/geo/query/acc.cgi?acc=GSE198473. Source data are provided with this paper.

## Source data

Source Data

## References

[1] Dammann O, Durum S, Leviton A (2001). Do white cells matter in white matter damage?. Trends Neurosci..

[2] Berger I, Peleg O, Ofek-Shlomai N (2012). Inflammation and early brain injury in term and preterm infants. Isr. Med. Assoc. J..

[3] Keil A (2010). Parental autoimmune diseases associated with autism spectrum disorders in offspring. Epidemiology.

[4] Atladottir HO, Schendel DE, Henriksen TB, Hjort L, Parner ET (2016). Gestational age and autism spectrum disorder: trends in risk over time. Autism. Res..

[5] Chaplin AB, Jones PB, Khandaker GM (2020). Association between common early-childhood infection and subsequent depressive symptoms and psychotic experiences in adolescence: a population-based longitudinal birth cohort study. Psychol. Med..

[6] Prinz M, Jung S, Priller J (2019). Microglia biology: one century of evolving concepts. Cell.

[7] Shemer A, Erny D, Jung S, Prinz M (2015). Microglia plasticity during health and disease: an immunological perspective. Trends Immunol..

[8] Schwabenland M (2021). Analyzing microglial phenotypes across neuropathologies: a practical guide. Acta Neuropathol..

[9] Paolicelli RC (2011). Synaptic pruning by microglia is necessary for normal brain development. Science.

[10] Schafer DP (2012). Microglia sculpt postnatal neural circuits in an activity and complement-dependent manner. Neuron.

[11] Cunningham CL, Martinez-Cerdeno V, Noctor SC (2013). Microglia regulate the number of neural precursor cells in the developing cerebral cortex. J. Neurosci..

[12] Hagemeyer N (2017). Microglia contribute to normal myelinogenesis and to oligodendrocyte progenitor maintenance during adulthood. Acta Neuropathol..

[13] Pont-Lezica L (2014). Microglia shape corpus callosum axon tract fasciculation: functional impact of prenatal inflammation. Eur. J. Neurosci..

[14] Schwarz JM, Bilbo SD (2012). Sex, glia, and development: interactions in health and disease. Horm. Behav..

[15] Bilbo SD (2005). Neonatal infection-induced memory impairment after lipopolysaccharide in adulthood is prevented via caspase-1 inhibition. J. Neurosci..

[16] McCarthy MM, Nugent BM, Lenz KM (2017). Neuroimmunology and neuroepigenetics in the establishment of sex differences in the brain. Nat. Rev. Neurosci..

[17] Stridh L (2013). Toll-like receptor-3 activation increases the vulnerability of the neonatal brain to hypoxia-ischemia. J. Neurosci..

[18] Patterson PH (2012). Maternal infection and autism. Brain Behav. Immun..

[19] Milligan R, Cockcroft K (2017). Working memory profiles in HIV-exposed, uninfected and HIV-infected children: a comparison with neurotypical controls. Front Hum. Neurosci..

[20] Nichols SL (2016). Learning and memory in children and adolescents with perinatal HIV infection and perinatal HIV exposure. Pediatr. Infect. Dis. J..

[21] Paus T, Keshavan M, Giedd JN (2008). Why do many psychiatric disorders emerge during adolescence?. Nat. Rev. Neurosci..

[22] Blank T, Prinz M (2013). Microglia as modulators of cognition and neuropsychiatric disorders. Glia.

[23] Das A (2017). RNA sequencing reveals resistance of TLR4 ligand-activated microglial cells to inflammation mediated by the selective jumonji H3K27 demethylase inhibitor. Sci. Rep..

[24] Waetzig V (2005). c-Jun N-terminal kinases (JNKs) mediate pro-inflammatory actions of microglia. Glia.

[25] Sousa C (2018). Single-cell transcriptomics reveals distinct inflammation-induced microglia signatures. EMBO Rep..

[26] Johnson MR (2015). Systems genetics identifies Sestrin 3 as a regulator of a proconvulsant gene network in human epileptic hippocampus. Nat. Commun..

[27] Awada R (2014). Autotaxin downregulates LPS-induced microglia activation and pro-inflammatory cytokines production. J. Cell Biochem.

[28] Broadbent NJ, Gaskin S, Squire LR, Clark RE (2010). Object recognition memory and the rodent hippocampus. Learn Mem..

[29] Celikel T (2007). Select overexpression of homer1a in dorsal hippocampus impairs spatial working memory. Front Neurosci..

[30] Cai Y (2018). Liver X receptor beta regulates the development of the dentate gyrus and autistic-like behavior in the mouse. Proc. Natl Acad. Sci. USA.

[31] Ransohoff RM, Perry VH (2009). Microglial physiology: unique stimuli, specialized responses. Annu Rev. Immunol..

[32] Sellner S (2016). Microglial CX3CR1 promotes adult neurogenesis by inhibiting Sirt 1/p65 signaling independent of CX3CL1. Acta Neuropathol. Commun..

[33] Otto G (2018). Synaptic nibbling. Nat. Rev. Neurosci..

[34] Alexopoulou L, Holt AC, Medzhitov R, Flavell RA (2001). Recognition of double-stranded RNA and activation of NF-kappaB by Toll-like receptor 3. Nature.

[35] Proost P (2003). Microbial Toll-like receptor ligands differentially regulate CXCL10/IP-10 expression in fibroblasts and mononuclear leukocytes in synergy with IFN-gamma and provide a mechanism for enhanced synovial chemokine levels in septic arthritis. Eur. J. Immunol..

[36] Ransohoff RM, Kivisakk P, Kidd G (2003). Three or more routes for leukocyte migration into the central nervous system. Nat. Rev. Immunol..

[37] Schoenborn JR, Wilson CB (2007). Regulation of interferon-gamma during innate and adaptive immune responses. Adv. Immunol..

[38] Mombaerts P (1992). RAG-1-deficient mice have no mature B and T lymphocytes. Cell.

[39] Silverman JL, Yang M, Lord C, Crawley JN (2010). Behavioural phenotyping assays for mouse models of autism. Nat. Rev. Neurosci..

[40] Rattazzi L (2013). CD4(+) but not CD8(+) T cells revert the impaired emotional behavior of immunocompromised RAG-1-deficient mice. Transl. Psychiatry.

[41] Fuchs J, Straub T, Seidl M, Kochs G (2019). Essential role of interferon response in containing human pathogenic bourbon virus. Emerg. Infect. Dis..

[42] Hatsuzawa K (2006). Involvement of syntaxin 18, an endoplasmic reticulum (ER)-localized SNARE protein, in ER-mediated phagocytosis. Mol. Biol. Cell.

[43] Setty SR (2007). BLOC-1 is required for cargo-specific sorting from vacuolar early endosomes toward lysosome-related organelles. Mol. Biol. Cell.

[44] Baasch S (2021). Cytomegalovirus subverts macrophage identity. Cell.

[45] Mohr CA (2010). A spread-deficient cytomegalovirus for assessment of first-target cells in vaccination. J. Virol..

[46] Datta M (2018). Histone deacetylases 1 and 2 regulate microglia function during development, homeostasis, and neurodegeneration in a context-dependent manner. Immunity.

[47] Schafer DP (2016). Microglia contribute to circuit defects in Mecp2 null mice independent of microglia-specific loss of Mecp2 expression. Elife.

[48] Patterson PH (2009). Immune involvement in schizophrenia and autism: etiology, pathology and animal models. Behav. Brain Res..

[49] Estes ML, McAllister AK (2016). Maternal immune activation: Implications for neuropsychiatric disorders. Science.

[50] Fernandez de Cossio L, Guzman A, van der Veldt S, Luheshi GN (2017). Prenatal infection leads to ASD-like behavior and altered synaptic pruning in the mouse offspring. Brain Behav. Immun..

[51] Meyer U (2014). Prenatal poly(i:C) exposure and other developmental immune activation models in rodent systems. Biol. Psychiatry.

[52] Hui CW (2018). Prenatal immune challenge in mice leads to partly sex-dependent behavioral, microglial, and molecular abnormalities associated with schizophrenia. Front. Mol. Neurosci..

[53] Tchessalova D, Tronson NC (2019). Memory deficits in males and females long after subchronic immune challenge. Neurobiol. Learn Mem..

[54] Missig G (2018). Perinatal immune activation produces persistent sleep alterations and epileptiform activity in male mice. Neuropsychopharmacology.

[55] Erickson MA (2018). Genetics and sex influence peripheral and central innate immune responses and blood-brain barrier integrity. PLoS One.

[56] Biesmans S (2013). Systemic immune activation leads to neuroinflammation and sickness behavior in mice. Mediators Inflamm..

[57] Smith CJ (2020). Neonatal immune challenge induces female-specific changes in social behavior and somatostatin cell number. Brain Behav. Immun..

[58] Nakaya M, Yamasaki M, Miyazaki Y, Tachibana H, Yamada K (2003). Estrogenic compounds suppressed interferon-gamma production in mouse splenocytes through direct cell-cell interaction. Vitr. Cell Dev. Biol. Anim..

[59] Nakaya M, Tachibana H, Yamada K (2006). Effect of estrogens on the interferon-gamma producing cell population of mouse splenocytes. Biosci. Biotechnol. Biochem.

[60] Bell MR (2018). Comparing postnatal development of gonadal hormones and associated social behaviors in rats, mice, and humans. Endocrinology.

[61] Traub S (2012). Sex bias in susceptibility to MCMV infection: implication of TLR9. PLoS One.

[62] Sonar SA, Shaikh S, Joshi N, Atre AN, Lal G (2017). IFN-gamma promotes transendothelial migration of CD4(+) T cells across the blood-brain barrier. Immunol. Cell Biol..

[63] Monteiro S, Roque S, Marques F, Correia-Neves M, Cerqueira JJ (2017). Brain interference: revisiting the role of IFNgamma in the central nervous system. Prog. Neurobiol..

[64] Saunders NR, Liddelow SA, Dziegielewska KM (2012). Barrier mechanisms in the developing brain. Front. Pharm..

[65] Ottum PA, Arellano G, Reyes LI, Iruretagoyena M, Naves R (2015). Opposing roles of interferon-gamma on cells of the central nervous system in autoimmune neuroinflammation. Front. Immunol..

[66] Filiano AJ (2016). Unexpected role of interferon-gamma in regulating neuronal connectivity and social behaviour. Nature.

[67] Yildirim-Buharalioglu G, Bond M, Sala-Newby GB, Hindmarch CC, Newby AC (2017). Regulation of epigenetic modifiers, including KDM6B, by interferon-gamma and interleukin-4 in human macrophages. Front. Immunol..

[68] Ivashkiv LB (2018). IFNgamma: signalling, epigenetics and roles in immunity, metabolism, disease and cancer immunotherapy. Nat. Rev. Immunol..

[69] Schaafsma W (2015). Long-lasting pro-inflammatory suppression of microglia by LPS-preconditioning is mediated by RelB-dependent epigenetic silencing. Brain Behav. Immun..

[70] Wendeln AC (2018). Innate immune memory in the brain shapes neurological disease hallmarks. Nature.

[71] Howe FS, Fischl H, Murray SC, Mellor J (2017). Is H3K4me3 instructive for transcription activation?. Bioessays.

[72] Goldmann T (2015). USP18 lack in microglia causes destructive interferonopathy of the mouse brain. EMBO J..

[73] Jordan S (2011). Virus progeny of murine cytomegalovirus bacterial artificial chromosome pSM3fr show reduced growth in salivary Glands due to a fixed mutation of MCK-2. J. Virol..

[74] Crawley JN (2007). Mouse behavioral assays relevant to the symptoms of autism. Brain Pathol..

[75] Dobin A (2013). STAR: ultrafast universal RNA-seq aligner. Bioinformatics.

[76] Liao Y, Smyth GK, Shi W (2014). featureCounts: an efficient general purpose program for assigning sequence reads to genomic features. Bioinformatics.

[77] Ewels P, Magnusson M, Lundin S, Kaller M (2016). MultiQC: summarize analysis results for multiple tools and samples in a single report. Bioinformatics.

[78] Murtagh F, Legendre P (2014). Ward’s hierarchical agglomerative clustering method: which algorithms implement Ward’s criterion?. J. Classif..

[79] Afgan E (2018). The Galaxy platform for accessible, reproducible and collaborative biomedical analyses: 2018 update. Nucleic Acids Res..

[80] Yao, Z. et al. A taxonomy of transcriptomic cell types across the isocortex and hippocampal formation. *Cell* **184**, 3222–3241.e26 (2021). 10.1016/j.cell.2021.04.021 PMC8195859 34004146

[81] Asp P (2011). Genome-wide remodeling of the epigenetic landscape during myogenic differentiation. Proc. Natl Acad. Sci. USA.

[82] Schwabenland M (2021). Deep spatial profiling of human COVID-19 brains reveals neuroinflammation with distinct microanatomical microglia-T-cell interactions. Immunity.

[83] Kaphzan H (2013). Genetic reduction of the alpha1 subunit of Na/K-ATPase corrects multiple hippocampal phenotypes in Angelman syndrome. Cell Rep..

[84] Quintana A (2012). Lack of GPR88 enhances medium spiny neuron activity and alters motor- and cue-dependent behaviors. Nat. Neurosci..

[85] Zhang HT (2002). Antidepressant-like profile and reduced sensitivity to rolipram in mice deficient in the PDE4D phosphodiesterase enzyme. Neuropsychopharmacology.

