# Supplementary material for: Neonatal immune challenge poses a sex-specific risk for epigenetic microglial reprogramming and behavioral impairment
Source: Nat Commun. 2023 May 11;14:2721. doi: 10.1038/s41467-023-38373-0 (PMC10175500; doi:10.1038/s41467-023-38373-0)

SUPPLEMENTARY FIGURES

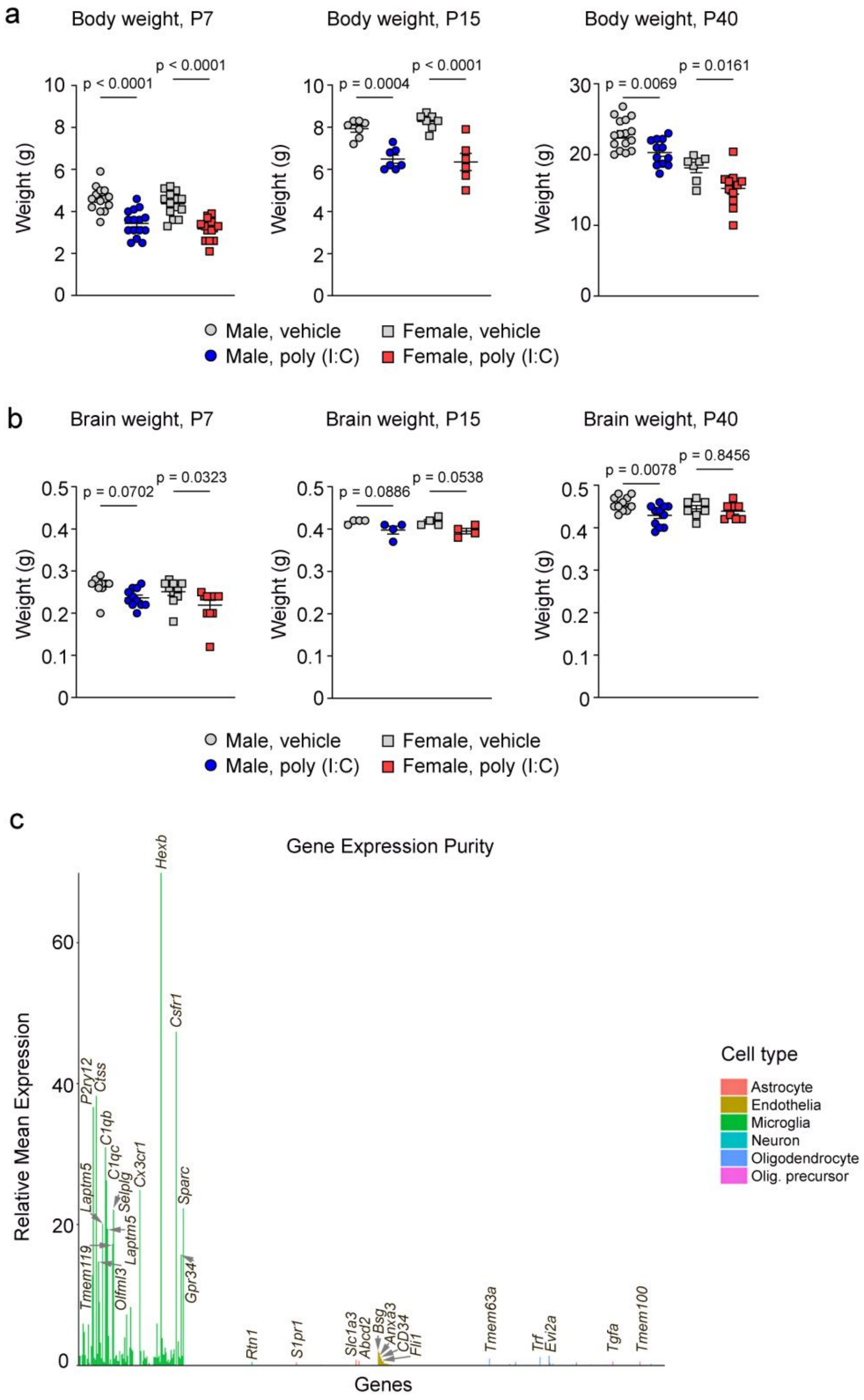

Supplementary Fig. 1: The body weight and brain weight of vehicle- and poly (I:C)-injected mice.
(a) The body weight and **(b)** the brain weight of male and female mice at P7, P15 and P40. Mice were injected with vehicle or poly (I:C) once daily starting from P2-P6. Data are presented as mean ± SEM. Each color-coded symbol represents data of an individual mouse (Body weight, P7: *n* = 14 for Male, vehicle; *n* = 15 for Male, Poly (I:C); *n* = 14 for Female, vehicle; *n* = 15 for Female, Poly (I:C). Body weight, P15: *n* = 7 for Male, vehicle; *n* = 7 for Male, Poly (I:C); *n* = 7 for Female, vehicle; *n* = 6 for Female, Poly (I:C). Body weight, P40: *n* = 16 for Male, vehicle; *n* = 12 for

Male, Poly (I:C); *n* = 7 for Female, vehicle; *n* = 11 for Female, Poly (I:C). Brain weight, P7: *n* = 10 for Male, vehicle; *n* = 11 for Male, Poly (I:C); *n* = 10 for Female, vehicle; *n* = 11 for Female, Poly (I:C). Brain weight, P15: *n* = 4 for Male, vehicle; *n* = 4 for Male, Poly (I:C); *n* = 4 for Female, vehicle; *n* = 4 for Female, Poly (I:C). Brain weight, P40: *n* = 11 for Male, vehicle; *n* = 11 for Male, Poly (I:C); *n* = 7 for Female, vehicle; *n* = 8 for Female, Poly (I:C)). Significant differences were determined by one-way ANOVA followed by Sidak multiple comparison test. P values are provided in the figure.

(c) Gene expression purity of microglial RNA-seq analysis displayed in Figure 1. Cell type-specific genes were obtained from McKenzie et al., 2018[1]. Representative gene names are indicated.

Source data are provided as a Source data file.

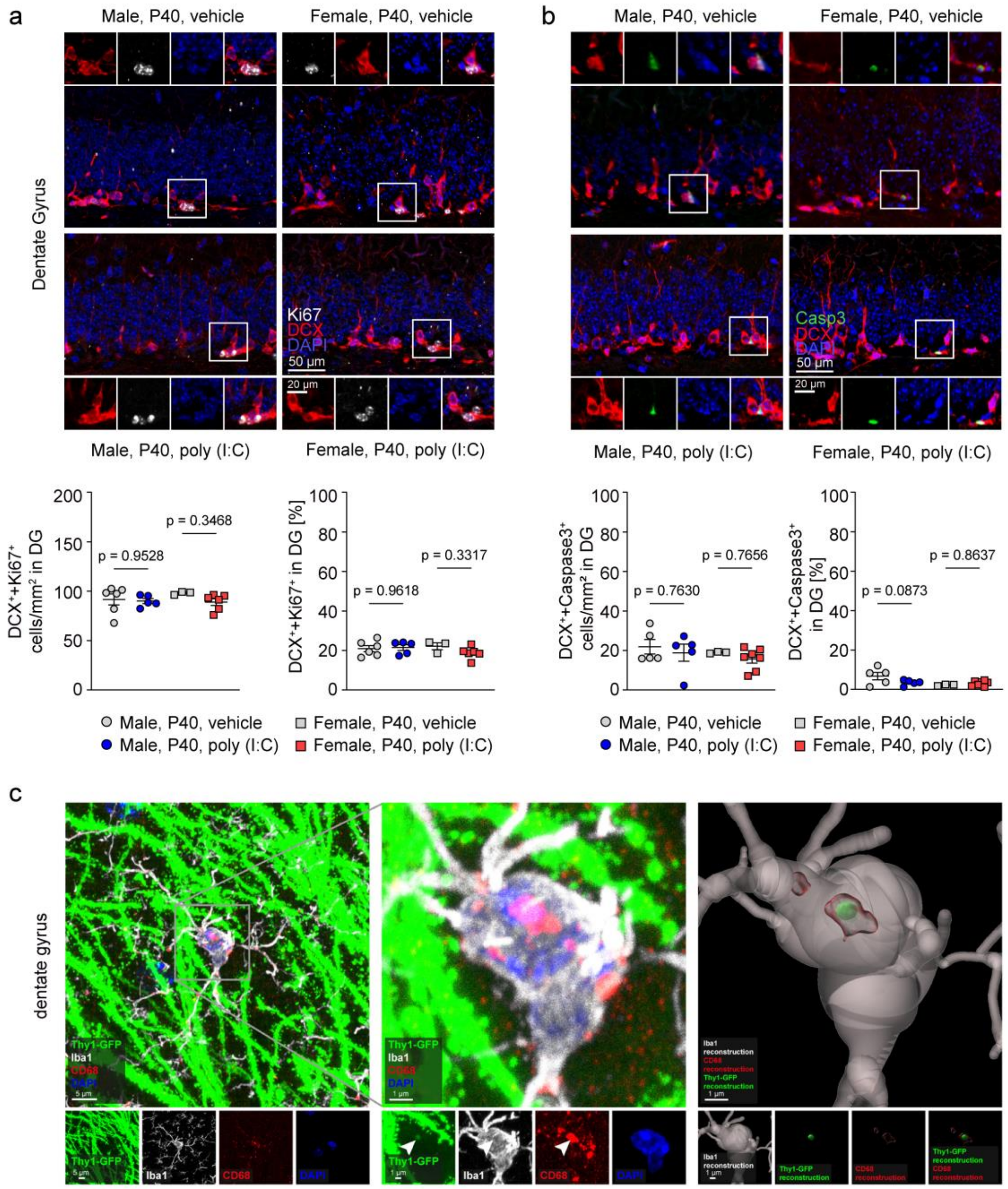

Supplementary Fig. 2: In the dentate gyrus of the hippocampus, cell proliferation and cell death of immature neurons remains unaltered after poly (I:C) challenge.

(a) Immunohistochemistry for Ki67 (white), DCX (red) and DAPI (blue) in the dentate gyrus of the hippocampus of poly (I:C) or vehicle injected P40 animals. Scale bar = 50 µm. Lower left panel: quantification of DCX^+ + $Ki67^+$ cells. Lower right panel: percentage DCX^+ + $Ki67^+$ cell number of total DCX^+ cell number. Data are presented as mean ± SEM. Each symbol represents an individual mouse (n = 6 for Male, P40, vehicle; n = 5 for Male, P40, Poly (I:C); n = 3 for Female, P40, vehicle; n = 6 for Female, P40, Poly (I:C)). Significant differences were determined by one-way ANOVA followed by Sidak multiple comparison test. P values are provided in the figure.

(b) Immunohistochemical detection of activated Caspase 3 (Casp3, green), DCX (red) and DAPI (blue) in the dentate gyrus of the hippocampus of poly (I:C)- or vehicle-injected P40 mice. Scale bar = 50 µm. Lower left panel: quantification of DCX^+ cells with a positive signal of activated Caspase 3. Lower right panel: percentage DCX^+ $Casp3^+$ cell number of total DCX^+ cell number. Data are presented as mean ± SEM. Each symbol represents an individual mouse (n = 5 for Male, P40, vehicle; n = 5 for Male, P40, Poly (I:C); n = 3 for Female, P40, vehicle; n = 7

for Female, P40, Poly (I:C)). Significant differences were determined by one-way ANOVA followed by Sidak multiple comparison test. P values are provided in the figure.

(c) Left panel: immunofluorescent image of Iba1 (white), CD68 (red) and DAPI (blue) in the dentate gyrus of a male poly (I:C)-injected Thy1-GFP (green) mouse. Scale bars represent 5 µm. The middle panel shows a higher magnification of the image on the left. GFP+ signal within CD68+ lysosomal structures that is disconnected from any neighboring neuronal processes is indicated by arrows in the single channels. Scale bars = 1 µm. The right panel shows the respective reconstruction. For exemplary purposes, only a fraction of the CD68-positive lysosomal compartment is reconstructed.

Source data are provided as a Source data file.

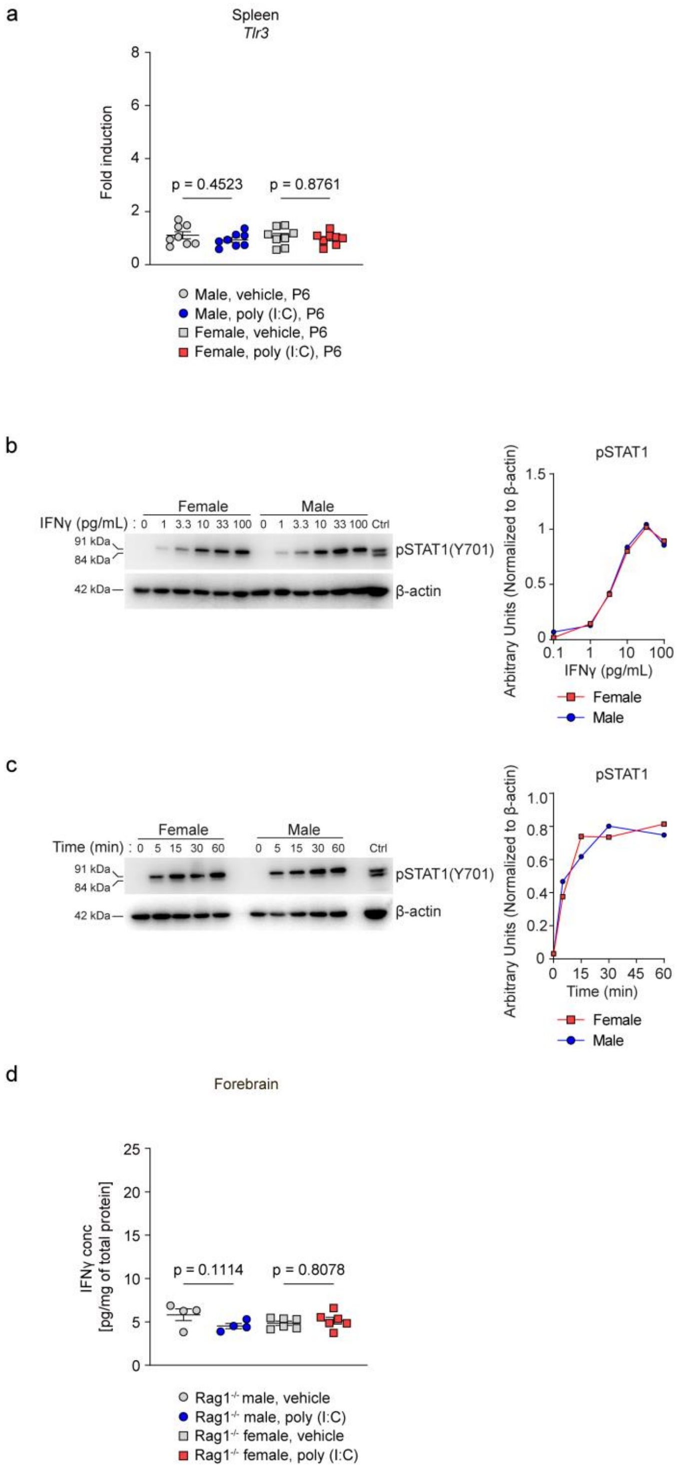

Supplementary Fig. 3: IFNγ response in male and female neonatal microglia *in vitro*.

(a) Quantitative RT-qPCR of spleen lysates obtained 3 hours after the last vehicle or poly (I:C) injection at postnatal day 6. Data are expressed as the ratio of *Tlr3* gene expression normalized to *Gapdh*. Data are presented as mean ± SEM. Each color-coded symbol represents data of an individual mouse (*n* = 8 for Male, vehicle, P6; *n* = 8 for Male, Poly (I:C), P6; *n* = 8 for Female, vehicle, P6; *n* = 8 for Female, Poly (I:C), P6). Significant differences were determined by one-way ANOVA followed by Sidak multiple comparison test. P values are provided in the figure.

(b) Dose-response of pSTAT1(Y701) of isolated neonatal microglia to IFNγ (1-100 ng/mL; 30 minutes). **(c)** Time course of STAT1 phosphorylation of isolated neonatal microglia to IFNγ (100 pg/mL; 5-60 minutes).

(d) IFNγ concentrations as measured by ELISA in forebrain lysates of *RAG1*$^{+/+}$ and *RAG1*$^{-/-}$ mice 7h after the last injection at postnatal day 6. Data are presented as mean ± SEM. Each color-coded symbol represents data of an

individual mouse (*n* = 4 for $Rag1^{-/-}$ male, vehicle; *n* = 4 for $Rag1^{-/-}$ male, poly (I:C); *n* = 4 for $Rag1^{-/-}$ female, vehicle; *n* = 4 for $Rag1^{-/-}$ female, poly (I:C)). Significant differences were determined by one-way ANOVA followed by Sidak multiple comparison test. P values are provided in the figure.

Source data are provided as a Source data file.

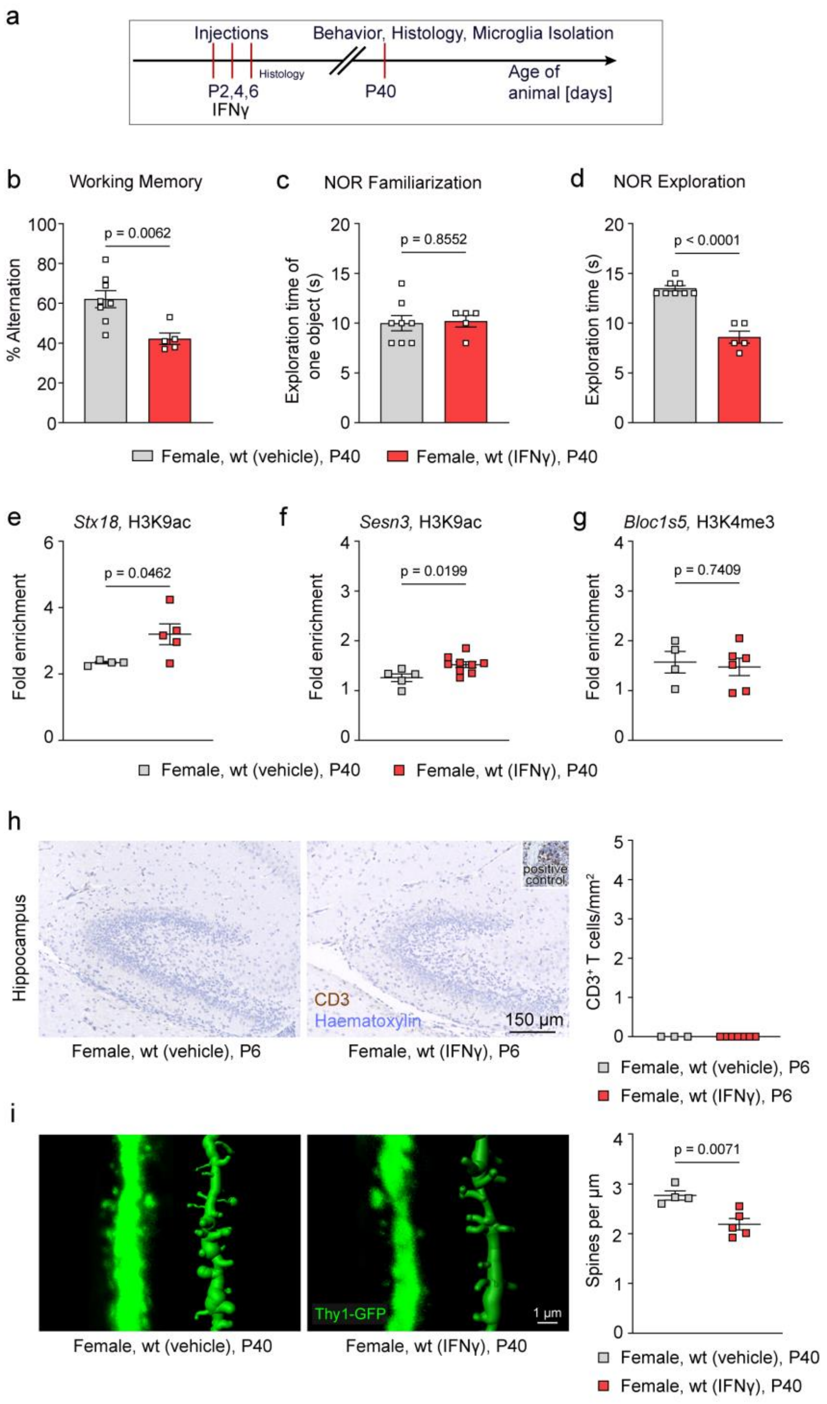

Supplementary Fig. 4: *In vivo* response to IFNγ-application in male and female mice.

(a) The schema illustrates the individual steps of the workflow to obtain the data shown in supplementary figures 4 b-i.

(b-d) Percentage alteration in a T-maze test (**b**), exploration time in a novel object recognition of one object (**c**) and of a novel object (**d**). Female mice were injected with vehicle or IFNγ on postnatal day 2, 4 and 6 and tested on postnatal day 40. Data are presented as mean ± SEM. Each dot represents data of an individual mouse *(n* = 8 for Female, wt (vehicle), P40; *n* = 5 for Female, wt (IFNγ), P40). Significant differences were determined by an unpaired, two-tailed t-test. The p value is provided in the figure.

(e-g) ChIP-qPCR analysis at P40 for H3K4me3 or H3K9ac occupancy on *Stx18, Sesn3 and Bloc1s5* promoters in microglia isolated from female mice, which were neonatally injected with vehicle or IFNγ. Quantification of enrichment is represented as fold-enrichment over the background level. Each color-coded symbol represents data

of an individual mouse *(Stx18*: *n* = 4 for Female, wt (vehicle), P40; *n* = 5 for Female, wt (IFNγ), P40). *Sesn3*: *n* = 5 for Female, wt (vehicle), P40; *n* = 9 for Female, wt (IFNγ), P40). *Bloc1s5*: *n* = 4 for Female, wt (vehicle), P40; *n* = 6 for Female, wt (IFNγ), P40)). Significant differences were determined by an unpaired, two-tailed t-test. P values are provided in the figures.

(h) Chromogenic staining for $CD3^+$ T cells (brown) and haematoxylin (blue) in the hippocampus of IFN□- or vehicle-injected female animals obtained at P6. Scale bar = 150 µm. Insert shows CD3-positive signals on spleen tissue as control. Each dot represents data of an individual mouse *(n* = 3 for Female, wt (vehicle), P6; *n* = 7 for Female, wt (IFNγ), P6).

(i) Fluorescent images of Thy1-GFP^+ cells in vehicle-treated and IFNγ-treated female animals. Representative 3D reconstructions of dendritic spines are shown next to the original histological images. Scale bar = 2 µm (left). Quantification of dendritic spines in the dentate gyrus is shown on the right. Data are presented as mean ± SEM. Each symbol represents data of one mouse *(n* = 4 for Female, wt (vehicle), P40; *n* = 5 for Female, wt (IFNγ), P40). Significant differences were determined by an unpaired, two-tailed t-test. The p value is provided in the figure.

Source data are provided as a Source data file.

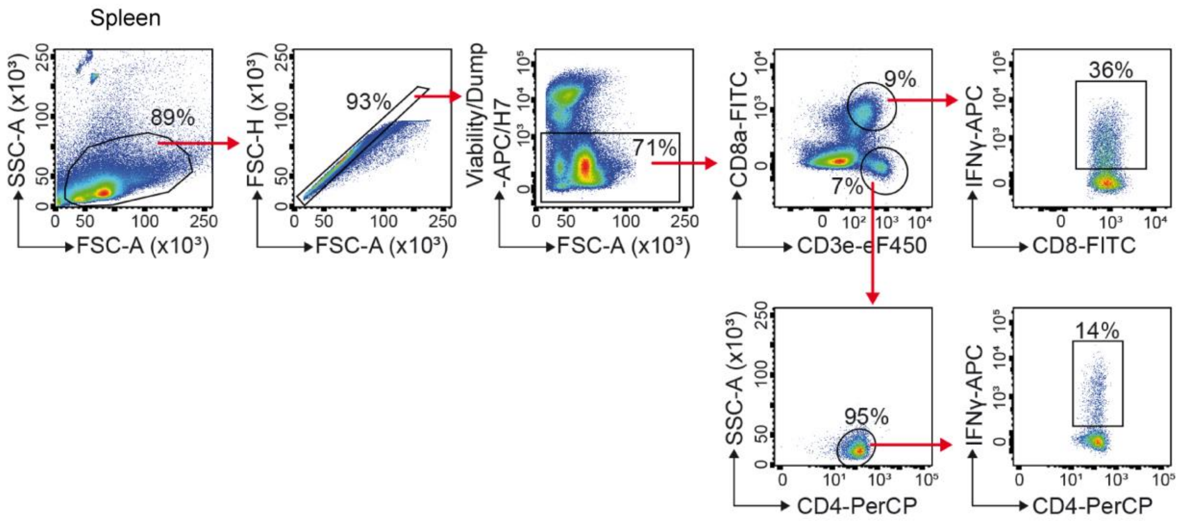

Supplementary Fig. 5: Gating strategy showing the analysis of T cell activation in mouse spleen. Female and male mice were infected with MCMV at P2 and P4 and sacrificed at day P15. Splenocytes were gated on live single cells gating and subsequently for CD3⁺, and CD4⁺ or CD8⁺ T cells. Events are plotted as CD4 versus IFNγ and CD8 versus IFNγ.

SUPPLEMENTARY REFERENCES

1 McKenzie, A. T. *et al.* Brain Cell Type Specific Gene Expression and Co-expression Network Architectures. *Sci Rep* **8**, 8868, doi:10.1038/s41598-018-27293-5 (2018).

UNCROPPED SCANS OF BLOTS

pSTAT1(Y701)

Supplementary Fig. 3b (upper blot) and Supplementary Fig. 3c (lower blot)

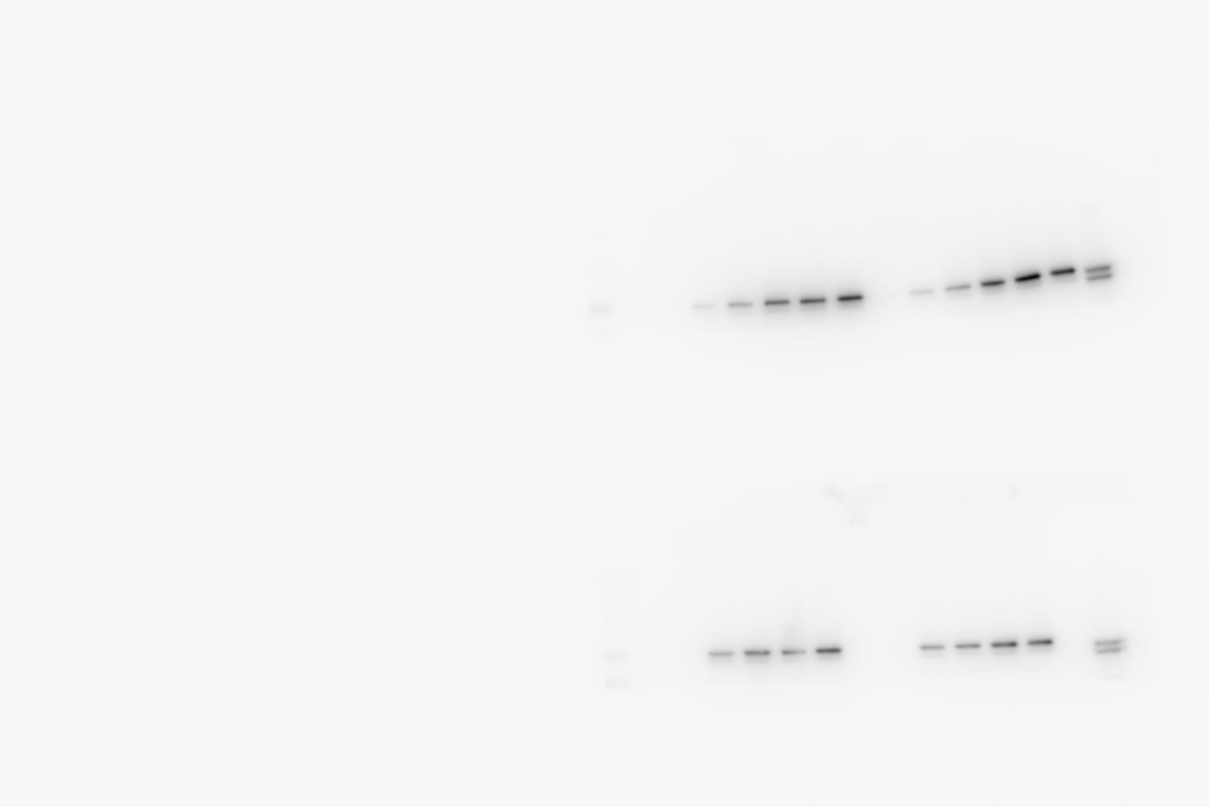

beta-Actin

Supplementary Fig. 3b (upper blot) and Supplementary Fig. 3c (lower blot)

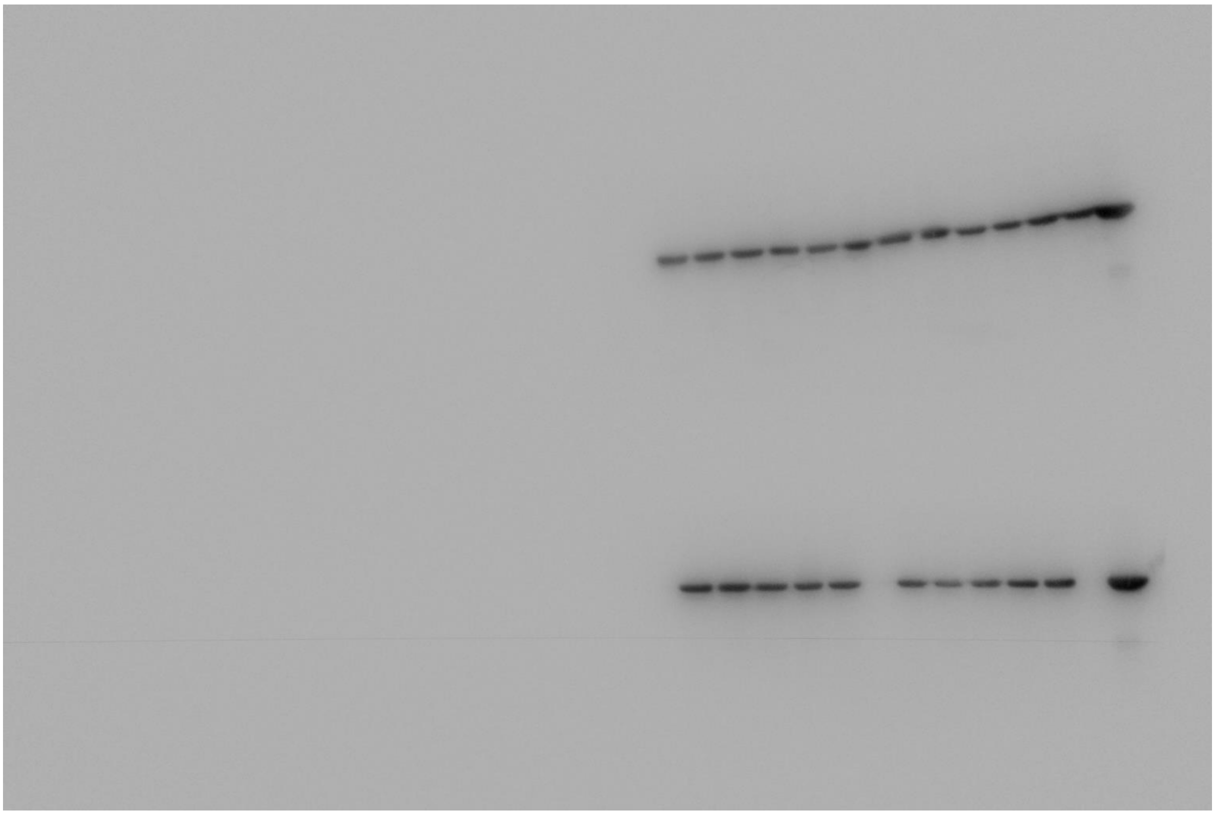

Supplement: Supplementary file 1 — Supplementary Information [file 41467_2023_38373_MOESM1_ESM.pdf]
