# Supplementary material for: Neonatal immune challenge poses a sex-specific risk for epigenetic microglial reprogramming and behavioral impairment
Source: Nat Commun. 2023 May 11;14:2721. doi: 10.1038/s41467-023-38373-0 (PMC10175500; doi:10.1038/s41467-023-38373-0)
Supplement: Supplementary file 2 — Description of Additional Supplementary Files [file 41467_2023_38373_MOESM2_ESM.pdf]

Description of Additional Supplementary Files

File Name: Supplementary Dataset 1

Description: Relative mean expression of cell type-specific genes indicating high purity of isolated cell populations used for RNA-seq analysis in Figure 1 (also see Supplementary Figure 1c). File Name: Supplementary Dataset 2 Description: Differentially expressed genes of sorted microglia cells obtained from male and female poly (I:C)- or vehicle-injected mice at postnatal days 7 and 40.
