# Supplementary material for: Neonatal immune challenge poses a sex-specific risk for epigenetic microglial reprogramming and behavioral impairment
Source: Nat Commun. 2023 May 11;14:2721. doi: 10.1038/s41467-023-38373-0 (PMC10175500; doi:10.1038/s41467-023-38373-0)
Supplement: Supplementary file 5 — Reporting Summary [file 41467_2023_38373_MOESM5_ESM.pdf]

nature portfolio

Corresponding author(s): Blank, Thomas

Last updated by author(s): Apr 14, 2023

Reporting Summary

Nature Portfolio wishes to improve the reproducibility of the work that we publish. This form provides structure for consistency and transparency in reporting. For further information on Nature Portfolio policies, see our Editorial Policies and the Editorial Policy Checklist.

Statistics

For all statistical analyses, confirm that the following items are present in the figure legend, table legend, main text, or Methods section.

n/a	Confirmed	
☐	☒	The exact sample size (*n*) for each experimental group/condition, given as a discrete number and unit of measurement
☐	☒	A statement on whether measurements were taken from distinct samples or whether the same sample was measured repeatedly
☐	☒	The statistical test(s) used AND whether they are one- or two-sided<br>*Only common tests should be described solely by name; describe more complex techniques in the Methods section.*
☒	☐	A description of all covariates tested
☒	☐	A description of any assumptions or corrections, such as tests of normality and adjustment for multiple comparisons
☐	☒	A full description of the statistical parameters including central tendency (e.g. means) or other basic estimates (e.g. regression coefficient) AND variation (e.g. standard deviation) or associated estimates of uncertainty (e.g. confidence intervals)
☐	☒	For null hypothesis testing, the test statistic (e.g. *F*, *t*, *r*) with confidence intervals, effect sizes, degrees of freedom and *P* value noted<br>*Give P values as exact values whenever suitable.*
☒	☐	For Bayesian analysis, information on the choice of priors and Markov chain Monte Carlo settings
☒	☐	For hierarchical and complex designs, identification of the appropriate level for tests and full reporting of outcomes
☒	☐	Estimates of effect sizes (e.g. Cohen's *d*, Pearson's *r*), indicating how they were calculated

Our web collection on statistics for biologists contains articles on many of the points above.

Software and code

Policy information about availability of computer code

Data collection	Library preparation and RNAseq were performed at the Genomics Core Facility "KFB - Center of Excellence for Fluorescent Bioanalytics" (University of Regensburg, Regenesburg, Germany; www.kfb-regensburg.de). The sequencing depth per sample was 30M reads. Imaging was performed on an Olympus Flouview 100 confocal laser scanning microscope (Olympus) using a 20x0.95 NA objective. Representative fluorescent pictures were taken with a BZ-9000 Biorevo microscope (Keyence).
Data analysis	Quality of sequencing reads stored in FASTQ files was assessed using FastQC (v0.67) and trimmed using Trim Galore! (v0.4.3). Reads were mapped on the mouse genome version mm10 (UCSC) using STAR aligner (v2.5.2) with RefGene annotation. The number of reads mapped to each gene (counts) was extracted from the BAM files using FeatureCount (v1.5.3) with the annotation version mm10 from UCSC and the following parameters: exon feature file, unstranded, a minimum mapping quality per read of 12, a minimum overlap of 1bp and other parameters set to default. A quality report of each step was generated using MultiQC (v1.5.0). R (v3.4.3) was used to perform the Ward error sum of squares hierarchical clustering method and principal components analyses (PCA). The process to extract the gene counts from FASTQ files was run on Galaxy. Using the DESeq2 model, the differentially expressed genes (DEGs) showing adjusted p-values (Wald test) of less than 0.05 and log 2 fold change greater than 0.97 were identified. Heatmaps were plotted using the pheatmap R package (v1.0.8) calculated from scaled (Z-scores), normalized read counts of DEGs with a hierarchical clustering of the rows. Pathway analysis was performed using Ingenuity Pathway Analysis (IPA, QIAGEN). Statistical analyses were performed using the Prism 9.5.1 (GraphPad) software; cells were reconstructed and analyzed using IMARIS software version 9.6.0 (Bitplane).

For manuscripts utilizing custom algorithms or software that are central to the research but not yet described in published literature, software must be made available to editors and reviewers. We strongly encourage code deposition in a community repository (e.g. GitHub). See the Nature Portfolio guidelines for submitting code & software for further information.

Data

Policy information about availability of data

All manuscripts must include a data availability statement. This statement should provide the following information, where applicable:

- Accession codes, unique identifiers, or web links for publicly available datasets
- A description of any restrictions on data availability
- For clinical datasets or third party data, please ensure that the statement adheres to our policy

Bulk RNA sequencing datasets were deposited into the Gene Expression Omnibus database under accession number GSE198473 and are available at the following URL: https://www.ncbi.nlm.nih.gov/geo/query/acc.cgi?acc=GSE198473.

Human research participants

Policy information about studies involving human research participants and Sex and Gender in Research.

Reporting on sex and gender	There were no human research participants involved in this study.
Population characteristics	There were no human research participants involved in this study.
Recruitment	There were no human research participants involved in this study.
Ethics oversight	There were no human research participants involved in this study.

Note that full information on the approval of the study protocol must also be provided in the manuscript.

Field-specific reporting

Please select the one below that is the best fit for your research. If you are not sure, read the appropriate sections before making your selection.

[x] Life sciences [ ] Behavioural & social sciences [ ] Ecological, evolutionary & environmental sciences

For a reference copy of the document with all sections, see nature.com/documents/nr-reporting-summary-flat.pdf

Life sciences study design

All studies must disclose on these points even when the disclosure is negative.

Sample size	Sample size is similar to sample sizes routinely used in the field for histological and behavioral comparisons (see Kaphzan et al. Cell Rep (2013), Quintana et al. Nat Neurosci (2012), Zhang et al. Neuropsychopharmacology (2002).)
Data exclusions	No data or mice were excluded.
Replication	If not indicated otherwise, all experiments were performed once.
Randomization	In all behavioral assays, subjects were randomly assigned to a group and the experiments were blind with respect to group assignments. Animals used for each experiment are littermates randomly assigned to the experimental groups.
Blinding	To obtain unbiased data, experimental mice of all relevant genotypes were processed together and cell quantifications were carried out blinded to the genotype.

Reporting for specific materials, systems and methods

We require information from authors about some types of materials, experimental systems and methods used in many studies. Here, indicate whether each material, system or method listed is relevant to your study. If you are not sure if a list item applies to your research, read the appropriate section before selecting a response.

Materials & experimental systems

n/a	Involved in the study
	☒ Antibodies
☒	☐ Eukaryotic cell lines
☒	☐ Palaeontology and archaeology
	☒ Animals and other organisms
☒	☐ Clinical data
☒	☐ Dual use research of concern

Methods

n/a	Involved in the study
☒	☐ ChIP-seq
	☒ Flow cytometry
☒	☐ MRI-based neuroimaging

Antibodies

Antibodies used

Iba1 (WAKO, polyclonal, cat# 019-19741)
DCX (Santa Cruz Biotechnology, clone C-18, cat# sc-8066)
Ki67 (Invitrogen, clone SolA15, cat# 14-5698-82)
CD68 (BioRad, clone FA-11, cat# MCA1957)
Cleaved Caspase-3 (Cell Signaling Technology, clone 5A1E, cat# 9664S)
Lamp2 (abcam, clone GL2A7, cat# ab13524)
Homer1 (Millipore, polyclonal, cat# ABN37)
vGlut1 (Synaptic Systems, polyclonal, cat# 135 304)
GAD67 (Sigma, clone K-87, cat# G5419-100UG)
CD3 (BioRad, clone CD3-12, cat# MCA1477)
Collagen IV (Merck, polyclonal, cat# AB769)
CD19 (BD Biosciences, clone 1D3, cat# 557655)
CD3e (eBioscience, clone 145-2C11, cat# 48-0031-82)
CD4 (BD Biosciences, clone RM4-5, cat# 553052)
CD8a (eBioscience, clone 53-6.7, cat# 11-0081-82)
NK1.1 (Biolegend, clone PK136, cat# 108714)
CD11a (eBioscience, clone M17/4, cat# 12-0111-82)
CD49d (Biolegend, clone R1-2, cat# 103603)
IFNgamma (eBioscience, clone XMG1.2, cat# 17-7311-82)
CD11b (eBioscience, clone M1/70, cat# 17-0112-83)
CD45 (eBioscience, clone 30-F11, cat# 12-0451-83)
Phospho-Stat1 (Cell Signaling Technology, clone 58D6, cat# 9167S)
β-Actin (Cell Signaling Technology, clone 8H10D10, cat# 3700S)
Donkey Anti-Rat IgG H&L (Alexa Fluor® 568) preadsorbed (Abcam, polyclonal, cat# ab175475)
Donkey anti-Goat IgG (H+L) Cross-Adsorbed Secondary Antibody, Alexa Fluor™ 488 (Invitrogen, polyclonal, cat# A-11055)
Donkey anti-Rabbit IgG (H+L) Highly Cross-Adsorbed Secondary Antibody, Alexa Fluor™ 647 (Invitrogen, polyclonal, cat# A-31573)
Donkey anti-Goat IgG (H+L) Cross-Adsorbed Secondary Antibody, Alexa Fluor™ 647 (Invitrogen, polyclonal, cat# A-21447)
Donkey anti-Rat IgG (H+L) Highly Cross-Adsorbed Secondary Antibody, Alexa Fluor™ 488 (Invitrogen, polyclonal, cat# A-21208)
Goat anti-Guinea Pig IgG (H+L) Highly Cross-Adsorbed Secondary Antibody, Alexa Fluor™ 647 (Invitrogen, polyclonal, cat# A-21450)
Donkey anti-Mouse IgG (H+L) Highly Cross-Adsorbed Secondary Antibody, Alexa Fluor™ 488 (Invitrogen, polyclonal, cat# A-21202)
Donkey anti-Rabbit IgG (H+L) Highly Cross-Adsorbed Secondary Antibody, Alexa Fluor™ 568 (Invitrogen, polyclonal, cat# A-10042)
Goat Anti-Rat IgG(H+L), Mouse ads-BIOT (SouthernBiotech, polyclonal, cat# 3050-08)

Validation

All antibodies used in this study have been validated by manufacturers or validated in published scientific literature. Validation data is available on the manufacturers' websites:
Iba1: https://labchem-wako.fujifilm.com/us/product/detail/W01W0101-1974.html
DCX: https://www.scbt.com/p/doublecortin-antibody-c-18
Ki67: https://www.thermofisher.com/antibody/product/Ki-67-Antibody-clone-SolA15-Monoclonal/14-5698-82
CD68: https://www.bio-rad-antibodies.com/monoclonal/mouse-cd68-antibody-fa-11-mca1957.html?f=purified
Cleaved Caspase-3: https://www.cellsignal.com/products/primary-antibodies/cleaved-caspase-3-asp175-5a1e-rabbit-mab/9664
Lamp2: https://www.abcam.com/products/primary-antibodies/lamp2-antibody-gl2a7-ab13524.html
Homer1: https://www.merckmillipore.com/DE/de/product/Anti-Homer1-Antibody,MM_NF-ABN37
vGlut1: https://www.sysy.com/product/135304
GAD67: https://www.sigmaaldrich.com/DE/de/product/sigma/g5419
CD3: https://www.bio-rad-antibodies.com/monoclonal/human-cd3-antibody-cd3-12-mca1477.html?f=purified
Collagen IV: https://www.merckmillipore.com/DE/de/product/Anti-Collagen-Type-IV-Antibody,MM_NF-AB769
CD19: https://www.bdbiosciences.com/en-de/products/reagents/flow-cytometry-reagents/research-reagents/single-color-antibodies-ruo/apc-cy-7-rat-anti-mouse-cd19.557655
CD3e: https://www.thermofisher.com/antibody/product/CD3e-Antibody-clone-145-2C11-Monoclonal/48-0031-82
CD4: https://www.bdbiosciences.com/en-de/products/reagents/flow-cytometry-reagents/research-reagents/single-color-antibodies-ruo/percp-rat-anti-mouse-cd4.553052
CD8a: https://www.thermofisher.com/antibody/product/CD8a-Antibody-clone-53-6-7-Monoclonal/11-0081-82
NK1.1: https://www.biolegend.com/en-us/products/pe-cyanine7-anti-mouse-nk-1-1-antibody-2840

CD11a: https://www.thermofisher.com/antibody/product/CD11a-LFA-1alpha-Antibody-clone-M17-4-Monoclonal/12-0111-82
CD49d: https://www.biolegend.com/en-us/products/biotin-anti-mouse-cd49d-antibody-437
IFNgamma: https://www.thermofisher.com/antibody/product/IFN-gamma-Antibody-clone-XMG1-2-Monoclonal/17-7311-82
CD11b: https://www.thermofisher.com/antibody/product/CD11b-Antibody-clone-M1-70-Monoclonal/17-0112-82
CD45: https://www.thermofisher.com/antibody/product/CD45-Antibody-clone-30-F11-Monoclonal/12-0451-83
Phospho-Stat1: https://www.cellsignal.com/products/primary-antibodies/phospho-stat1-tyr701-58d6-rabbit-mab/9167
β-Actin: https://www.cellsignal.com/products/primary-antibodies/b-actin-8h10d10-mouse-mab/3700
Donkey Anti-Rat IgG H&L (Alexa Fluor® 568) preadsorbed : https://www.abcam.com/products/secondary-antibodies/donkey-rat-igg-hl-alexa-fluor-568-preadsorbed-ab175475.html
Donkey anti-Goat IgG (H+L) Cross-Adsorbed Secondary Antibody, Alexa Fluor™ 488: https://www.thermofisher.com/antibody/product/Donkey-anti-Goat-IgG-H-L-Cross-Adsorbed-Secondary-Antibody-Polyclonal/A-11055
Donkey anti-Rabbit IgG (H+L) Highly Cross-Adsorbed Secondary Antibody, Alexa Fluor™ 647: https://www.thermofisher.com/antibody/product/A-31573.html
Donkey anti-Goat IgG (H+L) Cross-Adsorbed Secondary Antibody, Alexa Fluor™ 647: https://www.thermofisher.com/antibody/product/Donkey-anti-Goat-IgG-H-L-Cross-Adsorbed-Secondary-Antibody-Polyclonal/A-21447
Donkey anti-Rat IgG (H+L) Highly Cross-Adsorbed Secondary Antibody, Alexa Fluor™ 488: https://www.thermofisher.com/antibody/product/Donkey-anti-Rat-IgG-H-L-Highly-Cross-Adsorbed-Secondary-Antibody-Polyclonal/A-21208
Goat anti-Guinea Pig IgG (H+L) Highly Cross-Adsorbed Secondary Antibody, Alexa Fluor™ 647: https://www.thermofisher.com/antibody/product/Goat-anti-Guinea-Pig-IgG-H-L-Highly-Cross-Adsorbed-Secondary-Antibody-Polyclonal/A-21450
Donkey anti-Mouse IgG (H+L) Highly Cross-Adsorbed Secondary Antibody, Alexa Fluor™ 488: https://www.thermofisher.com/antibody/product/Donkey-anti-Mouse-IgG-H-L-Highly-Cross-Adsorbed-Secondary-Antibody-Polyclonal/A-21202
Donkey anti-Rabbit IgG (H+L) Highly Cross-Adsorbed Secondary Antibody, Alexa Fluor™ 568: https://www.thermofisher.com/antibody/product/Donkey-anti-Rabbit-IgG-H-L-Highly-Cross-Adsorbed-Secondary-Antibody-Polyclonal/A10042
Goat Anti-Rat IgG(H+L), Mouse ads-BIOT: https://www.southernbiotech.com/goat-anti-rat-igg-h-l-mouse-ads-biot-3050-08

Animals and other research organisms

Policy information about studies involving animals; ARRIVE guidelines recommended for reporting animal research, and Sex and Gender in Research

Laboratory animals	C57BL/6J mice were used as WT mice. RAG1-/- mice (strain no. 002216), IFNgamma-/- mice (strain no. 002287) and Thy1-GFP-M transgenic animals (strain no. 007788) all on C57BL/6J geneic background, were obtained from the Jackson Laboratories. The sex and age of the animals is provided in the figures and figure legends. Tissue obtained from a female 8 week old Cx3cr1cre x Usp18fl/fl mouse (Goldmann et al. EMBO 2015) served as a positive control for western blots.
Wild animals	The study did not involve wild animals.
Reporting on sex	Our study focuses on sex-specific effects upon neonatal immune stimulation. We have therefore analysed both male and female mice. Data is shown disaggregated for sex.
Field-collected samples	The study did not involve samples collected from the field.
Ethics oversight	Animal experiments were performed with the approval of the Regional Council of Freiburg, Germany, and the Research Ethics Committee at Leiden University, Leiden, Belgium. Experiments were carried out in accordance with the Fundamental Guidelines for Proper Conduct of Animal Experiment and Related Activities in Academic Research Institutions at the University of Freiburg, Germany.

Note that full information on the approval of the study protocol must also be provided in the manuscript.

Flow Cytometry

Plots

Confirm that:

- [x] The axis labels state the marker and fluorochrome used (e.g. CD4-FITC).
- [x] The axis scales are clearly visible. Include numbers along axes only for bottom left plot of group (a 'group' is an analysis of identical markers).
- [x] All plots are contour plots with outliers or pseudocolor plots.
- [x] A numerical value for number of cells or percentage (with statistics) is provided.

Methodology

Sample preparation	Spleens from naive and MCMV-infected mice were mechanically dissociated in Dissection Media and passed through a 100 um cell strainer. Red blood cells were lysed using RBC Cell Lysis buffer. Splenocates were counted on a hemocytometer and seeded in 5 ml FACS tubes at a density of 1,000,000 cells/mL and stimulated with PMA 50 ng/mL) and ionomycin (1 uM) for 3 hours. Cells were then collected by centrifugation and stained for FACS analysis.
Instrument	FACSAria III (Becton Dickinson)

Software	Data were acquired with FACSDiva software (Becton Dickinson). Data analysis was performed using FloJo software (version 10.5.3)
Cell population abundance	Cell population abundance is provided in the figure.
Gating strategy	Events were initially gated on FSC-A/SSC-A followed by doublet discrimination using FSC-A/FSC-H. Ded cells and lineage neative cells were excluded and T cells were gated using CD3, CD4 and CD8. Both CD3+CD3+ T cells and CD3+CD8+ T cells were gated on IFNg positive cells. All gates were determined using gluoresence minus one (FMO) controls.

☒ Tick this box to confirm that a figure exemplifying the gating strategy is provided in the Supplementary Information.
